# Cation Effects on the Acidic Oxygen Reduction Reaction at Carbon Surfaces

**DOI:** 10.1021/acsenergylett.3c02743

**Published:** 2024-03-01

**Authors:** J. L. Hübner, L. E. B. Lucchetti, H. N. Nong, D. I. Sharapa, B. Paul, M. Kroschel, J. Kang, D. Teschner, S. Behrens, F. Studt, A. Knop-Gericke, S. Siahrostami, P. Strasser

**Affiliations:** † Department of Chemistry, Chemical Engineering Division, Technical University of Berlin, 10623 Berlin, Germany; ‡ Centro de Ciências Naturais e Humanas, Federal University of ABC, Bairro Bangu, 09210-170 Santo André, Brazil; § Institute of Catalysis Research and Technology, Karlsruhe Institute of Technology, 76344 Eggenstein-Leopoldshafen, Germany; ∥ Department of Inorganic Chemistry, Fritz-Haber-Institute of the Max-Planck-Society, 14195 Berlin, Germany; ⊥ Department of Heterogeneous Reactions, Max-Planck-Institute for Chemical Energy Conversion, 45470 Mülheim an der Ruhr, Germany; # Department of Chemistry, Simon Fraser University, Burnaby, British Columbia V5A1S6, Canada

## Abstract

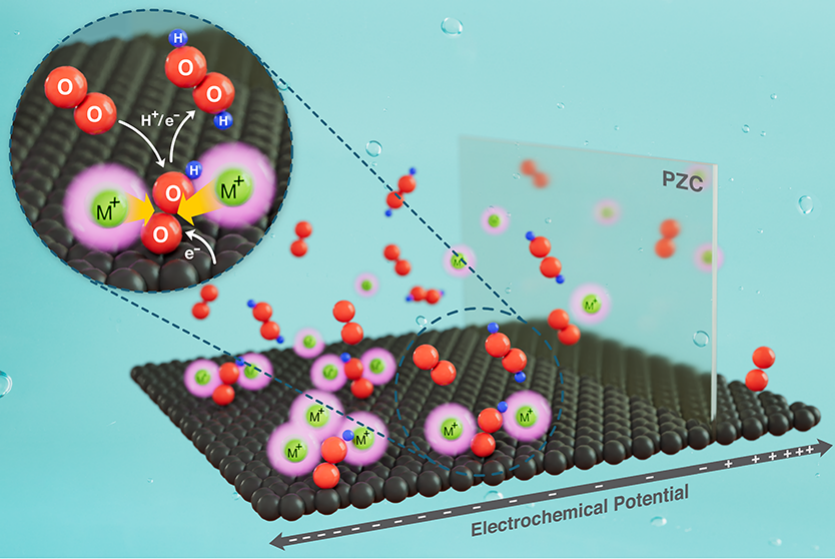

Hydrogen peroxide (H_2_O_2_) is a widely used green oxidant. Until now, research has focused on the development of efficient catalysts for the two-electron oxygen reduction reaction (2e^–^ ORR). However, electrolyte effects on the 2e^–^ ORR have remained little understood. We report a significant effect of alkali metal cations (AMCs) on carbons in acidic environments. The presence of AMCs at a glassy carbon electrode shifts the half wave potential from −0.48 to −0.22 V_RHE_. This cation-induced enhancement effect exhibits a uniquely sensitive on/off switching behavior depending on the voltammetric protocol. Voltammetric and *in situ* X-ray photoemission spectroscopic evidence is presented, supporting a controlling role of the potential of zero charge of the catalytic enhancement. Density functional theory calculations associate the enhancement with stabilization of the *OOH key intermediate as a result of locally induced field effects from the AMCs. Finally, we developed a refined reaction mechanism for the H_2_O_2_ production in the presence of AMCs.

Hydrogen peroxide (H_2_O_2_) is a powerful green oxidizing agent and due to its use in various fields, such as in the pulp and paper bleaching industry,^1^ chemical synthesis,^2,3^ and wastewater treatment^4,5^. It is one of the cornerstones of the chemical industry. The global annual consumption of H_2_O_2_ reached a value of 4.4 billion USD in 2023 with a compound annual growth rate (CAGR) of 4.4% in the forecast period of 2024–2032.^6^ The ever-growing need for H_2_O_2_ inspired research toward an alternative to the industrial anthraquinone process, which is currently producing 95% of the worldwide demand for H_2_O_2_. The process still suffers from high energy consumption, the generation of substantial amounts of organic byproducts, and risks associated with storage and extended transport routes of H_2_O_2_ stock solutions originating from the existence of only few centralized large scale anthraquinone process plants.^7,8^ These obstacles could be overcome by the electrochemical two-electron oxygen reduction process (2e^–^ ORR) for small-scale, on-site H_2_O_2_ production, where renewable energy sources can be applied as a power input:
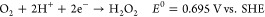
1

Acidic hydrogen peroxide solutions are of great significance in the chemical industry because of their superior oxidizing ability compared to alkaline solutions. Additionally, some reactions, e.g., the electro-Fenton process for the wastewater treatment, require acidic conditions in order to operate optimal (optimal performance within the pH range of 2.8–3.0^9^). Currently, only a limited number of expensive noble metal catalysts, such as platinum and palladium-based catalysts, have been identified as selective and stable for the 2e^–^ ORR in acid media.^10−12^ Lower cost carbon-based catalysts have shown reasonable H_2_O_2_ selectivities at low pH value but mainly at low current densities.^13−17^ Reports at higher industrially relevant current densities do exist,^18−21^ yet tend to place focus on conventional cell performance indicators and fail to address the role of the microenvironment of the interface. Driven by the ambition to address these limitations and to deploy the expanding understanding of atomic-scale interactions regarding the complex electrolyte effects on the activity of catalysts for the electrochemical carbon dioxide reduction reaction (CO_2_RR) and hydrogen evolution reaction (HER),^22−25^ scattered reports have appeared on pH effects^26,27^ or electrolyte composition^20,28−31^ on the 2e^–^ ORR. However, a molecular understanding of alkali metal cations (AMCs) effects on the H_2_O_2_ electrosynthesis, especially in acidic media, is far from complete. Most reports seem to ignore the key controlling role of the potential of zero change (PZC) of the electrified interface. The PZC is the electrode potential at which the interface has no free surface charge. The net charge of the ions at the interface is determined by the applied potential relative to the PZC. At potentials cathodic to the PZC, the electrode is negatively charged and attracts cations, while at more anodic potentials than the PZC, the electrode attracts anions. The position of the PZC and the applied working potential window determine, therefore, the composition of the electric double layer.

The primary objective of this study is to explore and understand the impact of AMCs in the electrolyte on the kinetics and thermodynamics of the reaction pathway of the 2e^–^ ORR toward H_2_O_2_ on carbon catalysts in strongly acidic conditions. Using experimental rotating ring disk surface voltammetry and time-resolved *in situ* X-ray photoemission spectroscopy (XPS) to track the accumulation of cations at electrified interfaces, in conjunction with density functional theory (DFT) calculations, we report, characterize, and unravel the mechanisms of the strong enhancement effect of AMCs on the 2e^–^ ORR.

In order to investigate the influence of AMCs, in particular K^+^, on the kinetics of the 2e^–^ ORR toward H_2_O_2_ in 0.1 M H_2_SO_4_, a rotating ring disk electrode (Pt ring, carbon disk RRDE) was employed (experimental details are given in Supporting Note 1). The smooth, polished glassy carbon (GC) disk was used as the catalyst material. As the PZC governs the surface charge of the electrode at any given applied potential, the experimental PZC of the GC disk was determined to have a value of around +0.3 V_RHE_ in 0.1 M H_2_SO_4_ (Figure S1). The Pt ring of the RRDE was held at constant +1.2 V_RHE_. Due to a steady sulfate anion adsorption and poisoning (Figure S2 and Supporting Note 2), the Pt-ring current and the H_2_O_2_ molar fraction selectivity (X) derived thereof can only be used as a qualitative probe for H_2_O_2_ formation.^32^ Repeated cleaning of the Pt ring by voltammetric pulses was not considered, since Pt dissolution and redeposition onto the GC had to be avoided.^33^ Importantly for this discussion, the 2e^–^ ORR voltammetric cycles were designed and performed with an upper turning potential (UTP) that was chosen either below or above the experimentally extracted PZC of the electrode.

*Surface voltammetry in the presence of AMCs below and above the PZC*. Figure 1a shows the time dependent anodic RRDE voltammetric potential scans of the carbon electrode during the 2e^–^ ORR to H_2_O_2_ in 0.1 M H_2_SO_4_ + 0.05 M K_2_SO_4_ with a UTP below the PZC. The production rates of the H_2_O_2_ formation, as indicated by the disk current density, monotonically increased with increasing cycling number. The decrease in the ring currents and the corresponding X with increasing cycling number is caused by the aforementioned Pt poisoning. However, a constant X within one cycle until the potential of ∼−0.5 V_RHE_ was observed, indicating no change in the ORR selectivity. The polarization curves stabilized after 190 cycles, when the characteristic 2e^–^ ORR voltammetric wave was fully apparent, which is coupled to the typical oxygen mass transport limiting range with a limiting current density of ∼−3 mA cm^–2^. The half wave potential of the 190th scan is −0.22 V_RHE_, whereas for the first scan, the potential for the same current density is −0.48 V_RHE_. Additionally, the onset potential (determined at a disk current density of 0.01 mA cm^–2^) shifted slightly from −0.01 mV (first scan) to 0.03 mV (190th scan). The corresponding Tafel slopes (Figure S3a) are −231 mV dec^–1^ for the first scan and −45 mV dec^–1^ for the 190th scan, reflecting an increase in catalytic reactivity, likely associated with lower kinetic barriers along the ORR reaction pathways. We hypothesize that the emerging microenvironment at the reactive surface during voltage cycling beneficially affects binding energies of reactive intermediates of the 2e^–^ ORR. Examples of such intermediates are oxygenated surface species, such as oxygen *O and peroxide intermediates *OOH (where * denotes an adsorption site).^34,35^ After cyclic voltammetry, SEM/EDX characterization of carbon surface revealed the formation of μm-sized K_2_SO_4_ crystals (Figure S4a), caused by salting out due to local accumulation of K^+^ cations passing the solubility limit of K_2_SO_4_ (111 g·l^–1^). Additionally, the voltammetric cycling exhibited a strong hysteresis behavior, with the anodic scan showing lower overpotentials for the 2e^–^ ORR than the cathodic scan (Figure S5). As corroborated further below, we attribute this to the fact that anodic scans originate at a more negative electrode potential where AMCs had more time to accumulate near the reactive electrocatalytic interface. This hypothesis is supported by the effect of scan rate on the location and width of the hysteresis behavior (Figure 1b). Slow scans reveal a wide hysteresis with low catalytic reactivity on the cathodic scan yet strong enhancement on the anodic scan. Counter to the expected voltammetric behavior of irreversible reactions and nonfaradaic capacitive effects, faster scans resulted in narrower hysteresis consistent with less time for cations to equilibrate their local distribution at very anodic or cathode electrode potentials. This observation points to the involvement of the K^+^ cations in the electric double layer (EDL) and the charge dependent equilibrium. Although, intercalation cannot be fully excluded as the prevalent origin of the gradual changes in the voltammetry. Intercalation and cointercalation seemed to be unlikely, as these phenomena were reported typically at much more negative potentials.^36,37^ Additionally, to the best of the authors’ knowledge, there are no reports of intercalation into glassy carbon at comparable conditions and/or reports of the effect of intercalation into glassy carbons on the ORR. Also, on/off switching of cation effects (Figure 1d) with the UTP speaks against intercalation of cations in the applied potential range. Finally, the influence of different K^+^ concentrations was investigated (Figure S5). With increasing K^+^ concentration, the time to reach constant H_2_O_2_ reactivity decreased. All investigated concentrations of K^+^ (0.1 M H_2_SO_4_ + 0.1/0.05/0.01 M K_2_SO_4_) ultimately resulted in a comparable 2e^–^ ORR activity enhancement. Figure 1c shows the analogous time-dependent anodic voltammetric scans during the 2e^–^ ORR in 0.1 M H_2_SO_4_ + 0.05 M K_2_SO_4_ with a UTP above the PZC. The initial 2e^–^ ORR activity was identical to the initial activity with a UTP < PZC, but in contrast to Figure 1a, no shift in the voltammetric wave, and hence electrocatalytic activity, was observed over time (see Tafel analysis in Figure S3b). The effect of the UTP is evidently striking. Postexperimental SEM/EDX characterization showed only small (<5 μm) droplets of K_2_SO_4_ on the surface (Figure S4b). To better understand this phenomenon, the reversibility of the AMC-induced activity enhancement was investigated (Figure 1d). After an initial 10 scans with a UTP < PZC, one single scan (11th) was performed with UTP > PZC (green lines), followed by consecutive three scans with a UTP < PZC. The expected catalytic enhancement during the first 10 scans was followed by an abrupt immediate annulling/reset of the catalytic improvement on the 11th scan (UTP > PZC), followed by a continuously rising catalytic enhancement during scans 12 to 14, with UTP < PZC. This on/off experiment highlights the role of the working potential window relative to the PZC. To date, the 2e^–^ ORR reactivity of carbon catalysts has been investigated at or above their PZC, which is why this cation surface accumulation effect has never been reported before utilizing RRDE techniques. Complementary to sweep voltammetry, a negative constant potential of −0.4 V_RHE_ led to improved H_2_O_2_ production rates in the presence of K^+^ cations yet no change in the ORR selectivity (Figure S6 and Supporting Note 3).

**Figure 1:**
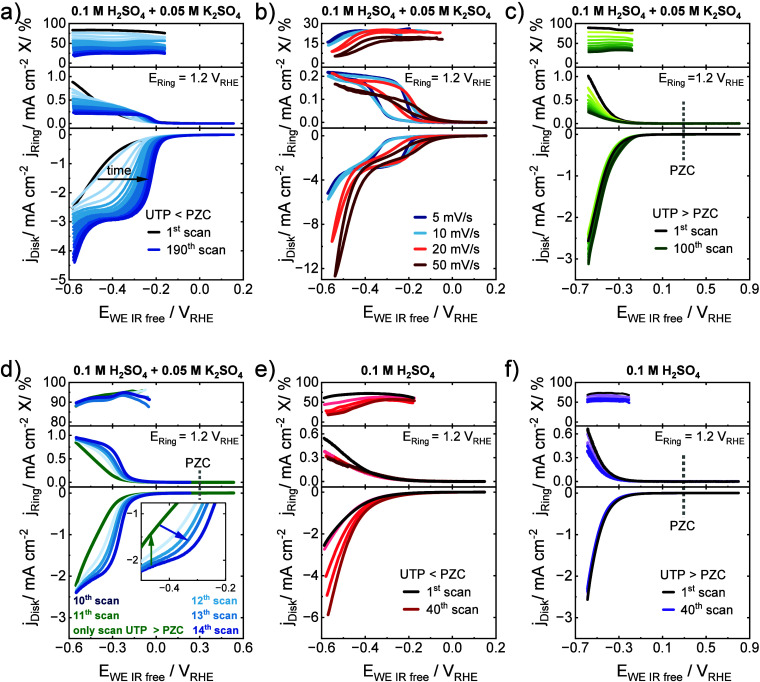
(a–d) Effect of K^+^ cations on the 2e^–^ ORR in 0.1 M H_2_SO_4_ + 0.05 M K_2_SO_4_ on GC at 1600 rpm: (a) Polarization curves for UTP below PZC. Time dependent activity improvement with first anodic scan (black) until constant activity after 190 scans (dark blue). Anodic scans with 5 mV/s in O_2_-saturated electrolyte are shown. (b) CVs at 5, 10, 20, and 50 mV/s after 190 scans at 5 mV/s. (c) Polarization curves for UTP above PZC. Initial activity shown in black, and 100th scans shown in dark green. Anodic scans with 5 mV/s in O_2_-saturated electrolyte are shown. (d) Anodic scans with 10th scan < PZC (dark blue), 11th scan > PZC (green), and 12–14th scan < PZC (light blue to blue), at 20 mV/s. Note: 1st to 9th scans below PZC are not shown. (e) Polarization curves for UTP below PZC in 0.1 M H_2_SO_4_ at 5 mV/s in O_2_-saturated electrolyte, 1st scan shown in black, and 40th scan shown in dark red. (f) Polarization curves for UTP above PZC in 0.1 M H_2_SO_4_ at 5 mV/s in O_2_-saturated electrolyte, the initial 1st scan shown in black and the 40th scan shown in violet.

*Surface voltammetry in absence of AMCs below and above the PZC*. In contrast to the sharp AMC-induced catalytic H_2_O_2_ production enhancement below the PZC, voltammetric cycling below the PZC in the absence of AMCs in the electrolyte (pure 0.1 M H_2_SO_4_) led to no catalytic enhancement in the H_2_O_2_ production associated with an oxygen mass transport limited current density in the applied potential range. The Tafel slopes remained around 210 mV dec^–1^ (Figure S3c). Although the catalytic onset potentials remained constant over time, the disk current densities <−0.4 V_RHE_ gradually increased. The ring current densities did not reflect the rise in disk current densities, and X decreased with more negative potentials. We therefore conclude a rise in the rate of the competing reactions under these conditions, such as the HER, the 4e^–^ ORR to water, or the H_2_O_2_ reduction reaction (H_2_O_2_RR). Efforts to use physicochemical SEM/EDX maps of the carbon surface after voltammetry to get insight in the origin of the voltammetric behavior remained inconclusive (Figure S7 and Supporting Note 4). Finally, Figure 1f shows the anodic voltammetric scans for a UTP above the PZC in absence of AMCs in the electrolyte. Similar to Figure 1c, no catalytic enhancement was evident over time. Here, the disk current densities remained almost constant over the entire potential range. Similar Tafel slopes of −209 and −221 mV dec^–1^ were found for the 1st and 100th scans for a UTP above the PZC indicating an insignificant effect on the reaction kinetics (Figure S3d). In order to investigate the effect of AMCs on the HER, we performed voltammetric scans under Ar-saturated conditions with and without K^+^ cations in the electrolyte. In agreement with the literature,^20^ a suppression of the HER due to the presence of K^+^ cations was confirmed (Figure S8). The suppression was stronger if a UTP below the PZC was applied. However, due to the low disk current densities under Ar-saturated conditions, it can be concluded that the HER only plays a minor role within the applied potential windows.

*In situ XPS in the K 2p core level range*. To directly track the accumulation of AMCs at the reactive carbon interface, time-resolved *in situ* XPS measurements were carried out, utilizing a previously reported membrane-electrode flow cell.^38^ The goal of these measurements was the direct observation of the potential controlled accumulation and repulsion of K^+^ cations at and from the carbon electrode surface (trilayer graphene on Nafion-N117). Details of the carbon electrode fabrication and cell configuration are provided in Supporting Note 5. In the first set of measurements, the carbon electrode surface was divided into a given number of distinct spots, which were cycled through and measured in consistent order. Survey spectra were taken before and after the electrochemical protocol (Figure S9). The potential protocol involved an applied electrode potential step and was held at +0.49 V_RHE_ for 4.5 h, followed by a potential step and holding at −0.61 V_RHE_ for 6 h, followed by a return to +0.49 V_RHE_ for 4 h. A number of spectra were recorded during each potential step.

Figure 2a shows a selection of nine high-resolution *in situ* XPS spectra of the carbon interface in the K 2p core-level region at the three applied electrode potentials (0.49, −0.61, 0.49 V_RHE_ from left to right) each at three selected measurement times (time increase from bottom to top) for an identical spot. Figure S10 shows the corresponding spectra in their full binding energy range, containing both the K 2p and C 1s core level regions. Due to the overlap of C 1s (carbon atoms bonded to Nafion F atoms) and K 2p(3/2), deconvolution was necessary to quantitatively analyze the temporal evolution of the K content of the sample. Figure 2b shows the overlaid spectra for each time. Figure 2a,b evidences that at +0.49 V_RHE_, there is no accumulation of K^+^ cations near the carbon interface over 4.5 h. By contrast, at −0.61 V_RHE_, the K 2p core level peaks gradually increase in intensity with time, suggesting the accumulation of K^+^ cations at the interface. After returning to +0.49 V_RHE_, the K 2p peak intensities decreased, pointing to the slow migration of K^+^ cations away from the interface. The K 2p core level binding energy appeared to be potential dependent (± ∼0.7 eV). The expected core level shift for K^+^ cations outside the EDL is −1.1 eV (based on the ±1.1 V bias), whereas for K^+^ cations in direct contact with the grounded graphene electrode, no shift is expected. Therefore, the position of the K^+^ cations inside the EDL can be assumed. Since the core level shift is independent of the hold time at −0.61 V_RHE_, it is most likely not caused by the formation of K_2_SO_4_ crystals. Another origin for the core level shift would be a change in the oxidation state of potassium (e.g., reduction of K^+^ to K^0^). However, in the applied potential range, it is not possible to reduce K^+^, as the standard redox potential of K^+^/K^0^ is −2.93 V. Figure 2c shows the K/(C + K) molar ratios, averaged over all measured spots of the sample (detailed ratios for each spot are shown in Figure S11). To exclude an accidental onset of K^+^ cations accumulation after 4.5 h at 0.49 V_RHE_ and to validate that the K^+^ cation accumulation can be triggered solely by shifting the electrode potential, a second experiment with a shorter 30 min initial hold potential at 0.49 V_RHE_ was conducted (Figure S12). The K 2p core level peaks increased as soon as −0.61 V_RHE_ was applied, proving that the accumulation of K^+^ cations at the cathode is largely influenced by the applied potential and not by the hold time. The surprisingly slow time scale of the K^+^ cation accumulation and repulsion can be attributed to the fact that K^+^ cations have to cross the entire Nafion membrane before they will enter the membrane-carbon interface. Also, as the carbon catalyst faces the vacuum region, this may lead to a relatively dry membrane-catalyst interface, which might slow down solvent assisted ion migration. Conversely, once the K^+^ cations have passed the membrane entering the carbon electrode interface they may get trapped there during interfacial charge reversal.

**Figure 2:**
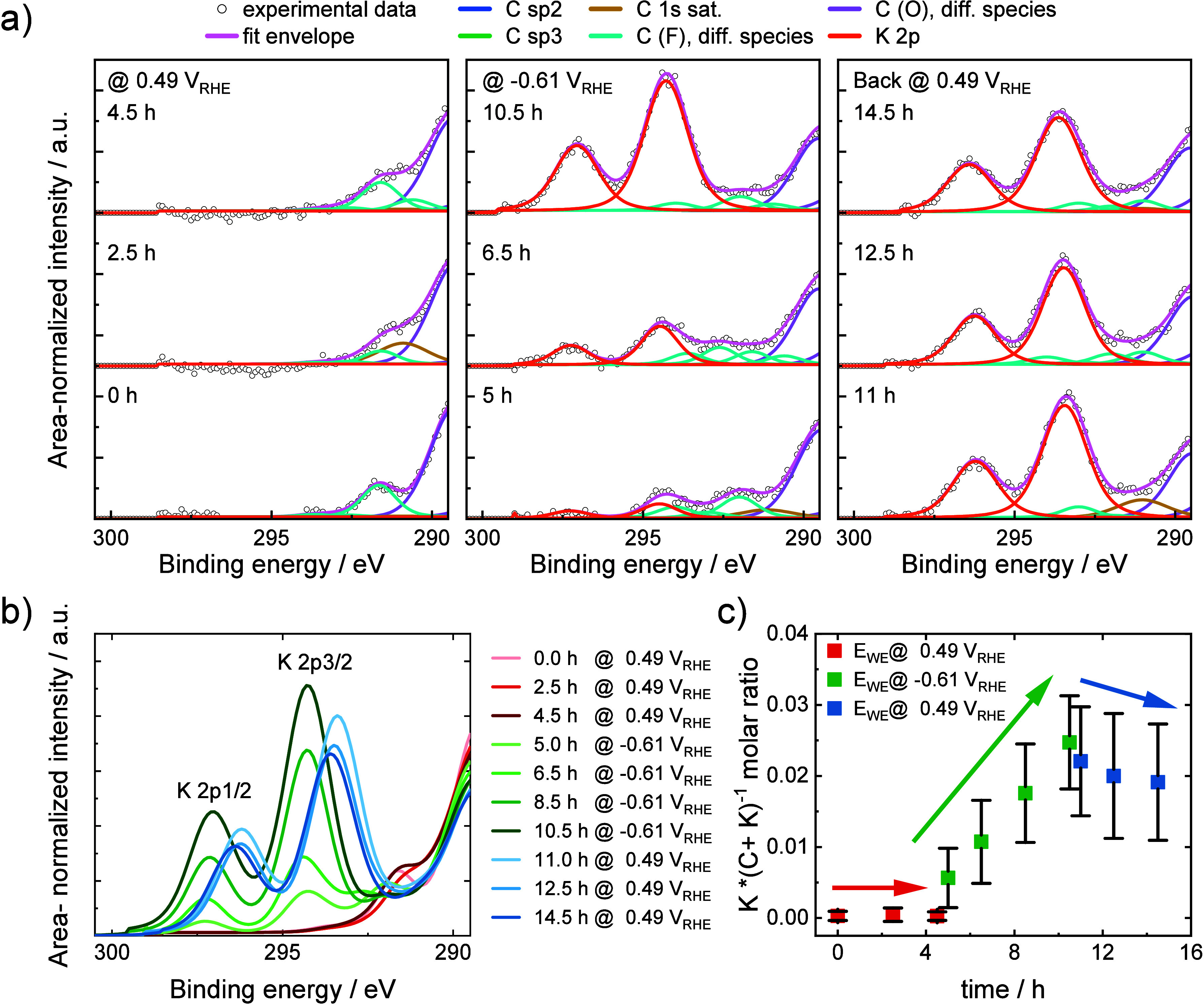
(a) XPS spectra of trilayer graphene on Nafion-N117 in the K 2p region over time at one selected spot and different potentials. Spectra were acquired at a pass energy of 20 eV and an excitation energy of 1000 eV. (b) Stacked area-normalized intensities of the K 2p peak for same spot as in (a) over time. (c) Averaged K 2p/(C 1s + K 2p) molar ratio of all measured spots over time as a function of applied potential.

*Density functional theory (DFT) calculations*. To substantiate and explain the experimental findings, DFT calculations were carried out with the Quantum ESPRESSO^39^ simulation package, utilizing GGA-PBE functionals to describe the exchange-correlation energy^40^ and SSSP pseudopotentials to account for the core electrons.^41^ For more computational details, the reader is referred to the computation details section in the Supporting Note 6. Previous reports show the presence of the cation effect on different reactions using a variety of different approaches. For instance, Zhang et al. found that alkali metal cations in acidic electrolytes can improve the H_2_O_2_ electrogeneration significantly utilizing an electrolyzer unit.^20^ In their work, *ab initio* molecular dynamics (AIMD) simulation was employed to show that Na^+^ cations adsorb on the electrode surface and create a local coordination environment that drives H^+^ atoms away from the surface and therefore reduces the H_2_O_2_RR. However, the authors noted that it is not possible to observe the same cation effect applying the RRDE technique due to the strong agitation and the low steady-state surface concentration of alkali metal cations at the electrode surface. Resasco et al. investigated the cation effect for the CO_2_RR and employed constrained minima hopping calculations to determine the solvation shell of different AMCs.^42^ The authors noted that larger solvated cations are more energetically favored at the outer Helmholtz plane, which results in a higher cation coverage for these species, thus increasing the local field strength on the adsorbates.^42^ Herein, we delve into the local field effect induced by cations on the 2e^–^ ORR intermediates, by modeling it through an explicitly applied field.^42−44^ The theoretical calculations show the stabilization of the ORR intermediates, in terms of the Gibbs free energy difference, when the local field effect is considered, as can be seen in Figure 3. The effect is similar among the different possible active sites in glassy carbon (all investigated active sites can be found in Figure S13 and the coordinates of the optimized surface models in Table S1), with a decrease in Δ*G* for all reaction intermediates following the increase in the local field.^43,44^ The *OOH intermediate is widely considered to be the key intermediate of the 2e^–^ ORR and can be therefore used as an activity descriptor.^34,35^ This analysis shows that the distance between the *OOH intermediate and the catalytic surface remains the same. However, under a positive local field induced by large cations such as K^+^, the hydrogen atom turns toward the solution and away from the surface. These geometric changes also contribute to the additional energy gain observed in the ΔG_*OOH_ values, resulting in a decrease of ΔG_*OOH_ of up to ∼−0.8 eV considering a positive applied field. Figure 4 shows the activity volcano plot for the 2e^–^ ORR. Without any applied field (hollow symbols), the selected defects and functional groups lie on the weak *OOH binding region of the volcano plot, which is in agreement with the literature.^45^ The strength of the electric field originating from solvated K^+^ cations in the vicinity of the catalyst surface was, depending on the size of the graphene flake and therefore the K^+^ surface coverage, calculated to be in the range of 0.77−1.44 V/Å (ORCA,^46−48^ PBE/def2-TZVP, and BHHLYP/def2-TZVP, details of the calculations are given in Supporting Note 7, Supporting Note 7, Figure S14, Tables S2−S8). Applying a value of 0.60 V/Å results in a shift toward the top of the volcano (full symbols) for three of the considered active sites (defects: 555–777, 55–77, and oxygen functional group: Vac-O), which is also in agreement with the experimental observations and with literature.^42−44^ This shift is enough to account, from a qualitative perspective, for the changes observed in the half wave potential, from −0.48 V to −0.22 V_RHE_, when K^+^ cations are added to the electrolyte (Figure 1a). Considering these results, we propose a new reaction mechanism by which the key reaction intermediate, *OOH, is stabilized by the positive local field induced by AMCs, such as K^+^, acting as Lewis acids (eqs 2 and 3):
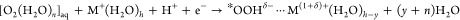
2
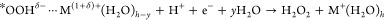
3

**Figure 3:**
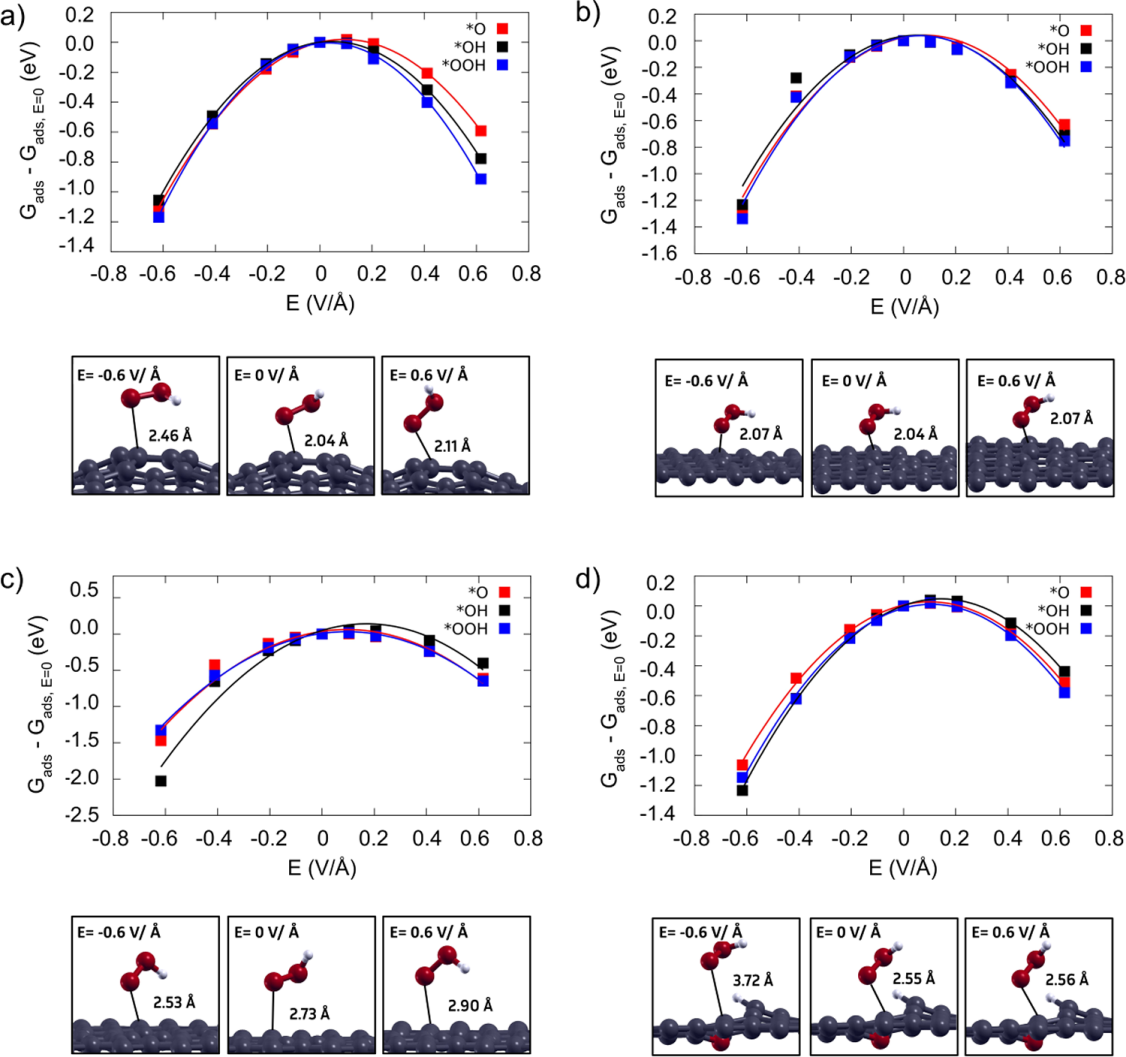
Field effect on ORR intermediates and the optimized geometry of the *OOH intermediate for different carbon defects and functional groups: 555–6–777 (a), 555–777 (b), 55–77 (c), and vac-O, carbon vacancy (d).

**Figure 4:**
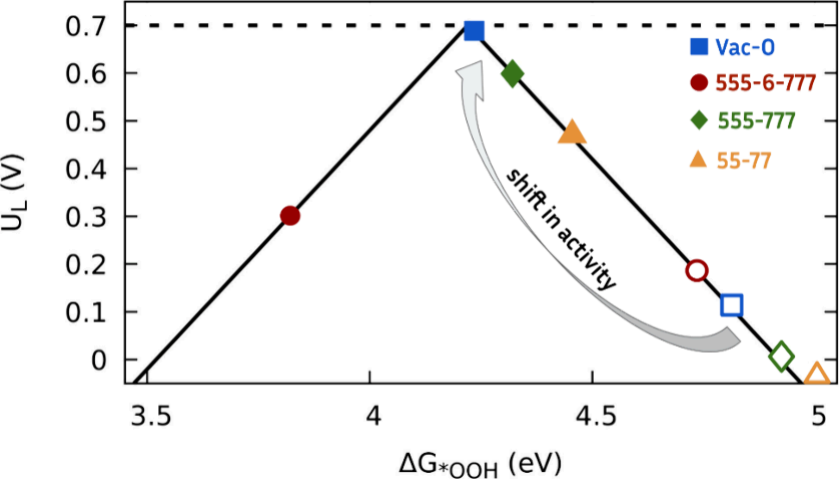
Activity volcano plot for the 2e^–^ ORR to H_2_O_2_ with the limiting potential (*U*_L_) as a function of ΔG_*OOH_. A black dotted line represents the equilibrium potential for this reaction (0.695 V_RHE_). The arrow indicates the shift in activity when the local induced field changes from *E* = 0 V/Å (hollow symbols) to 0.6 V/Å (full symbols).

In conclusion, this contribution has combined theory and experiments to explore the significant cation-induced electrocatalytic enhancement effects on the 2e^–^ ORR toward H_2_O_2_ in acidic solutions, by focusing on K^+^ cations as an example. We showed and explored the cation-induced enhancements in a RRDE environment, where the catalytic enhancement during voltammetric cycling showed a strong dependence on the UTP relative to the PZC. Due to the enhancement effect, a H_2_O_2_-selective voltammetric wave with mass transport limited current consistent with a 2e^–^ transfer process emerged. The half wave potential was shifted anodically from −0.48 to −0.22 V_RHE_. Time-resolved *in situ* XPS measurements visualized the potential-depended enrichment and repulsion of K^+^ cations from the working electrode surface. Finally, we were able to explain the experimentally observed cation effects using DFT calculations, whereby we were able to formulate a new reaction mechanism for the 2e^–^ ORR by the cation-induced stabilization of the key reaction intermediate, the *OOH intermediate. The presented insights into the significant influence of the electrolyte on the electrocatalytic 2e^–^ ORR in acidic conditions pave the way for a commercialization of the process utilizing cheap carbon catalysts and could also trigger future work on other promising 2e^–^ ORR catalyst systems.

## Supporting Information Available

Additional experimental details of chemicals, materials, sample preparation, and electrochemical characterization; assessment of Pt-ring poisoning; determination of PZC; additional SEM and elemental mapping images; additional electrochemical activity measurement; computational details (PDF)
