# Supplementary material for: Cation Effects on the Acidic Oxygen Reduction Reaction at Carbon Surfaces
Source: ACS Energy Lett. 2024 Mar 1;9(4):1331–8. doi: 10.1021/acsenergylett.3c02743 (PMC11019649; doi:10.1021/acsenergylett.3c02743)
Supplement: Supplementary file 1 — nz3c02743_si_001.pdf [file nz3c02743_si_001.pdf]

Supporting Information

Cation Effects on the Acidic Oxygen Reduction Reaction at Carbon Surfaces

J. L. Hübner[a], L. E. B. Lucchetti[b,c], H. N. Nong[a,*], D. I. Sharapa[d], B. Paul[a], M. Kroschel[a], J. Kang[a], D. Teschner[e,f], S. Behrens[d], F. Studt[d], A. Knop-Gericke[e,f], S. Siahrostami[b,g], P. Strasser[a,*]

[a] Department of Chemistry, Chemical Engineering Division, Technical University of Berlin, 10623 Berlin, Germany

[b] Department of Chemistry, Simon Fraser University, Burnaby, British Columbia V5A1S6, Canada

[c] Centro de Ciências Naturais e Humanas, Federal University of ABC, Bairro Bangu, 09210-170 Santo André, Brazil

[d] Institute of Catalysis Research and Technology, Karlsruhe Institute of Technology, 76344 Eggenstein-Leopoldshafen, Germany

[e] Department of Inorganic Chemistry, Fritz-Haber-Institute of the Max-Planck-Society, 14195 Berlin, Germany

[f] Department of Heterogeneous Reactions, Max-Planck-Institute for Chemical Energy Conversion, 45470 Mülheim an der Ruhr, Germany

[g] Department of Chemistry, University of Calgary, Calgary, Alberta T2N1N4, Canada

*Email: hong.n.nong-reier@campus.tu-berlin.de

*Email: pstrasser@tu-berlin.de

Supporting Note 1: Experimental details

Chemicals

For the preparation of the electrolytes H_2SO_4 (Ultrapur sulphuric acid 96 %, Supelco), K_2SO_4 (Potassium sulphate ≥ 98 % crystalline, Carl Roth), were used without further purification. All solutions containing K_2SO_4 were prepared using Milli Q water, whereas H_2SO_4 solutions without additionally metal cations were prepared using Ultrapur water (Supelco). Easy transfer trilayer graphene on a polymer film (TLG, model: G/G/G/P-10-10) was purchased from Graphenea (San Sebastian, Spain). Ethyl acetate (Supelco) was used to remove the polymer layer on top of the TLG. Nafion-NR212 and N117 (purchased from Ion Power GmbH) were used as membranes for the H-cell and in situ XPS measurements respectively.

Catalyst characterisation

To characterize the glassy carbon (GC) surface a JSM-7401F scanning electron microscope (SEM) from JEOL was utilized. A secondary electron detector with an acceleration voltage of 10 kV was used. EDX spectra and mapping were obtained using an acceleration voltage of 16 kV.

Electrochemical characterisation by RRDE technique

The 2-electron oxygen reduction reaction ($2e^-$ORR) tests were performed in a homemade glass H-cell with a leakage free silver /silver chloride (Ag/AgCl) (Innovative Instruments, Inc.) or a reversible hydrogen electrode (RHE, Gaskatel) reference electrode and a graphite counter electrode. 0.1 M H_2SO_4 and 0.1 M H_2SO_4 + x K_2SO_4 (x = 0.01, 0.05 and 0.1 M) were used as an electrolyte. 0.1 M H_2SO_4 was chosen as an initial electrolyte instead of the more commonly used $HClO_4$, due to the desire to produce acidic H_2O_2 solutions utilizing high current densities in full electrolyzers units which requires non-toxic and stable electrolytes. All experiments were conducted using a rotating ring disk electrode (RRDE) (Pine Research, model number: E7R9) with a glassy carbon disk (0.2475 cm^2) and a platinum ring (0.1866 cm^2). The reference electrodes were regularly calibrated to RHE (H_2 gas over freshly cleaned Pt surface) in each electrolyte and the data was corrected accordingly. The cathode and anode compartment were separated by an Nafion-NR212 membrane. In order to minimize the influence of contaminations from outside the H-cell (e.g., abrasion from the rotator) a strong overflow of 1 L/min air or N_2 gas was applied. During the reaction either O_2 or Ar- gas was constantly supplied to the

bottom part of the cell (10 mL/min). The RRDEs were polished with an 0.05 µm aluminium oxide polishing suspension (Buehler) and cleaned in an ultra-sonication bath with isopropanol and ultrapure water. The electrochemical tests were controlled by a Biologic SP 150 in combination with the EC-Lab software. All potentials were reported vs RHE scale and manually IR corrected using equation S.1.

Equation S1. IR-Free RHE potential

$$E_{RHE} = E_{Ref} + E_{Ag/AgCl} + R * I \qquad \text{(S1)}$$

E_{RHE}: Potential vs RHE/ V

E_{Ref}: Applied potential versus reference electrode / V

$E_{Ag/AgCl}$: Potential of reference electrode measured against RHE / V

R: Ohmic resistance / Ω

I: Current / A

The Ohmic resistance was obtained by galvanostatic electrochemical impedance spectroscopy (GEIS) at 0 mA. The frequency of the sine function used in the study was in the range 1 to 100000 Hz, and the amplitude was set to 100 µA.

To quantify the selectivity of the reduction towards the $2e^-$ ORR, the H_2O_2 molar fraction selectivity was applied and calculated accordingly to Equation 2:

Equation S2. H_2O_2 molar fraction selectivity

$$X = \frac{2I_R/N}{I_D + I_R/N} \qquad \text{(S2)}$$

X: H_2O_2 molar fraction

I_R: Ring current / A

I_D: Disk current / A

N: Collector efficiency (37 % provided by Pine Research)

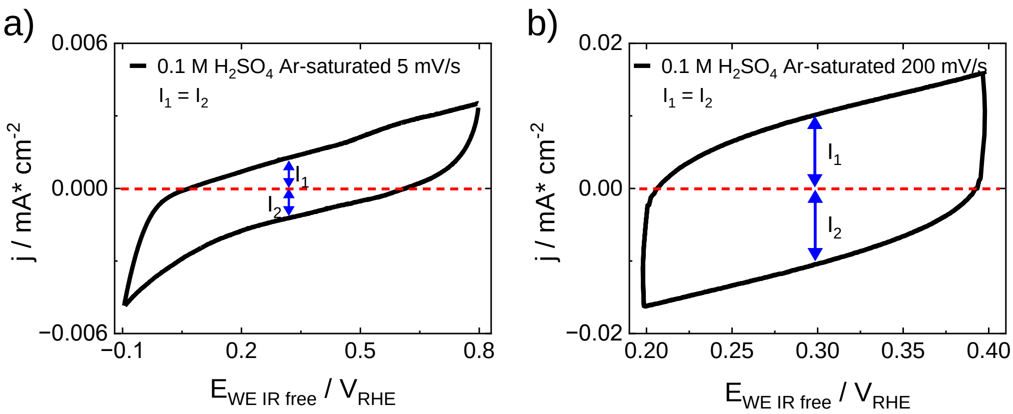

Figure S1: Determination of the potential of zero charge (PZC) of GC: Exemplary cyclovoltammetry in capacitive region of glassy carbon in 0.1 M H_2SO_4 at **a)** 5 mV/s and **b)** 200 mV/s in Ar-saturated electrolyte at 1600 rpm. At pure capacitive behavior with no faradaic charge PZC is located at $I_{cathodic}$ = I_{anodic}, dashed red line at I =0 mA. The PZC of GC in 0.1 M H_2SO_4 and 0.1 M H_2SO_4 + 0.05 M K_2SO_4 was determined to be 0.3 and 0.315 V_{RHE} correspondingly.

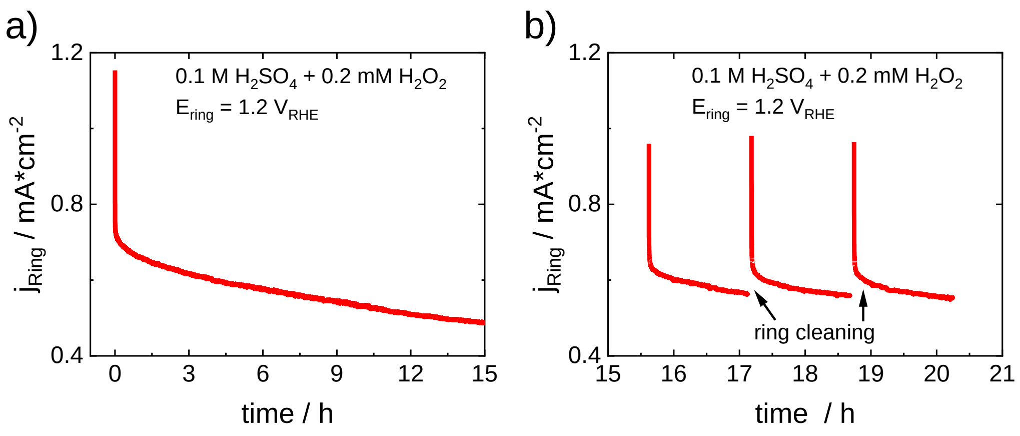

Figure S2: Pt- ring poisoning of RRDE electrode under constant potential in 0.1 M H_2SO_4 + 0.2 mM H_2O_2 at 1600 rpm **a)** without ring cleaning **b)** with ring cleaning in between.

Supporting Note 2:

Figure S2 a) and b) show the Pt- ring poisoning of the RRDE electrode under a constant potential of 1.2 V_{RHE} over time at 1600 rpm. Prior each experiment the Pt-ring was cleaned while cycling between 0.060 and 1.5 V_{RHE} in 0.1 M H_2SO_4 (Ar -saturated) until the characteristic Pt- features were reached. Afterwards the electrolyte was exchanged to a solution of 0.1 M H_2SO_4 + 0.2 mM H_2O_2, without exposing the cleaned electrode to atmosphere. In total 5 L of 0.1 M H_2SO_4 + 0.2 mM H_2O_2 were circulated during the experiment, using a peristaltic pump. Therefore, a constant concentration of H_2O_2 can be assumed during the experiment. Additionally, the concentration of H_2O_2 before and after the experiment was determined by the spectrophotometric analysis with titanium oxysulfate.[1] Only a negligible change in the H_2O_2 concentration was found. Figure S2 a) shows the ring current density j_{ring} after applying a constant potential of 1.2 V_{RHE} over 15.5 h. The decrease in j_{ring} with time can be attributed to anion poisoning, which in turn decreases the collector efficiency for the oxidation of H_2O_2.[2] After additional ring cleaning (30 cycles. 200 mV/s, 0.06- 0.76 V_{RHE}) the j_{ring} increased drastically (Figure S2 b)). Nevertheless, the authors chose not to include additional ring cleaning in the main experiments (Figure 1 a-f)), in order to avoid electrodeposition of Pt on the GC. Identical trends were observed in 0.1 M H_2SO_4 +0.05 M K_2SO_4 + 0.2 mM H_2O_2.

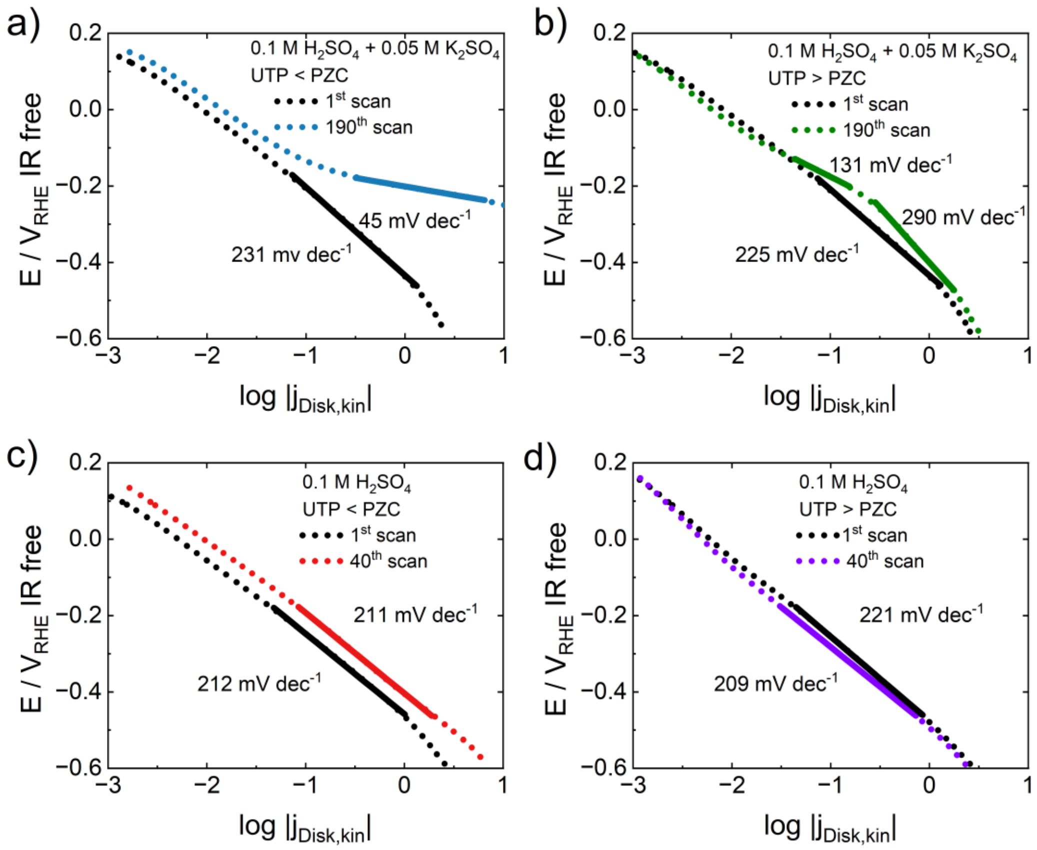

Figure S3: Corresponding Tafel plots for Figure 1 first and last scans (2e⁻ ORR at GC 1600 rpm, 5 mV/s, O_2 -saturated electrolyte): **a)** 0.1 M H_2SO_4 + 0.05 M K_2SO_4, upper turning potential (UTP) < PZC **b)** 0.1 M H_2SO_4 + 0.05 M K_2SO_4, UTP > PZC **c)** 0.1 M H_2SO_4, UTP < PZC **d)** 0.1 M H_2SO_4, UTP > PZC.

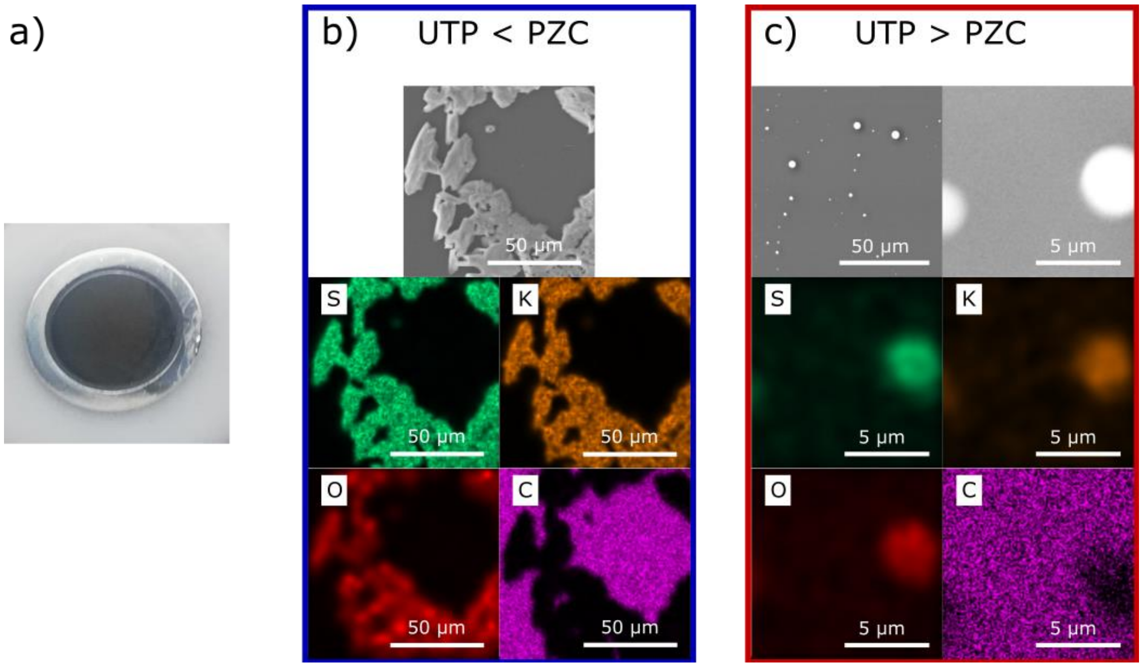

Figure S4: a) Picture of RRDE after 17h with UTP below PZC in 0.1 M H_2SO_4 + 0.05 M K_2SO_4. **b)** SEM/ EDX images of GC shown in a). **c)** SEM/ EDX images of GC after 17h with UTP above PZC in 0.1 M H_2SO_4 + 0.05 M K_2SO_4. RRDE tip was blow dried in N_2 directly after both electrochemical experiments.

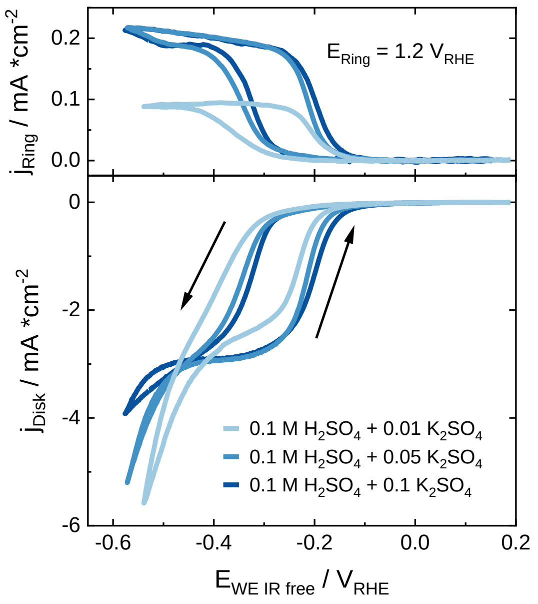

Figure S5: Effect of the different K^+ concentrations on $2e^-$ ORR in 0.1 M H_2SO_4 at GC at 1600 rpm and 5 mV/s. CVs after constant activity was reached for 0.1 M H_2SO_4 + x K_2SO_4 with x= 0.1, 0.05 and 0.01 M.

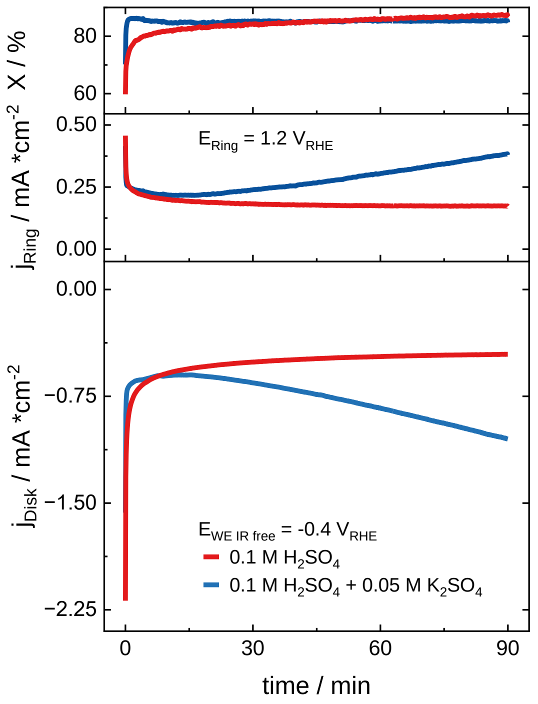

Figure S6: 2e$^-$ ORR in O_2 -saturated 0.1 M H_2SO_4 and 0.1 M H_2SO_4 + 0.05 M K_2SO_4 on GC at $E_{WE\ IR\ free}$ = -0.4 V_{RHE} and 1600 rpm.

Supporting Note 3:

While applying a constant disk potential of -0.4 V_{RHE} the cationic enhancement effect on the 2e$^-$ ORR is apparent. In 0.1 M H_2SO_4 the absolute value of the resulting currents decrease with time, whereas in 0.1 M H_2SO_4 + 0.05 M K_2SO_4 the absolute value of the resulting currents in the first minute's decrease strongly and later increase steadily. Interestingly, resulting in similar X in 0.1 M H_2SO_4 and 0.1 M H_2SO_4 + 0.05 M K_2SO_4, implying higher H_2O_2 production rates in the presents of K^+ cations but a constant ORR selectivity. Please note, that the value of the X can be misleading due to the accumulation of H_2O_2 caused by a partial oxidation at the Pt-ring (starting with a collector efficiency of 37 % but decreasing due to Pt poisoning) and the Pt-ring poisoning (Supporting Note 2) itself. To account for the H_2O_2 accumulation in the cyclovoltammetry experiments the authors performed a background subtraction of the ring current in the region where no disk current was detected. This approach is not applicable for chronoamperometry.

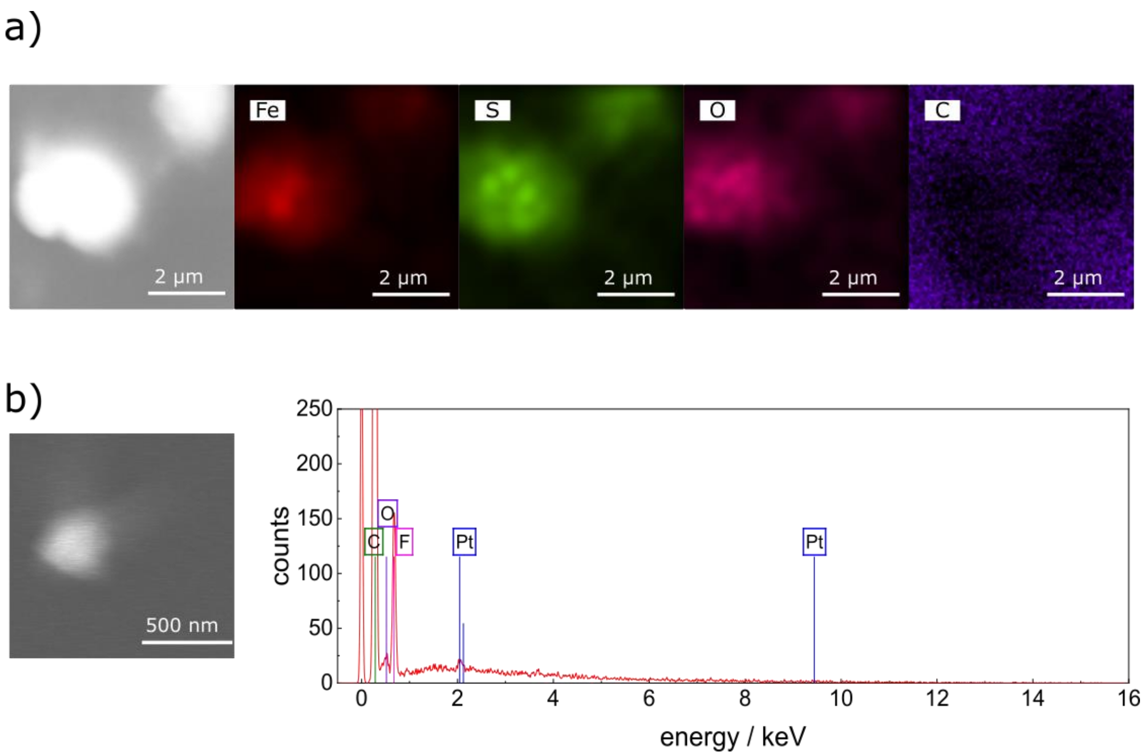

Figure S7: a) SEM/ EDX mapping of Fe particles **b)** SEM image/ EDX spectra of Pt particle on GC after electrochemistry with UTP below PZC in 0.1 M H_2SO_4. RRDE tip was blow dried in N_2 directly after electrochemical experiment.

Supporting Note 4:

With and without the presence of K^+ metal cations, small Fe and Pt particles (100 nm – 1 µm) were found. The origin of the iron contamination is likely to be the threads of the RRDE and the inner parts of the RRDE tips which are made of stainless steel. Pt particles could originate from dissolution of the Pt-ring and the consequently electrodeposition of Pt-particles on the GC electrode. Although, it is difficult to distinguish between Pt particles from electrodeposition and Pt abrasion from the polishing procedure of the electrodes, since Pt particles were found before and after electrochemistry. However, since the experiments were repeated multiple times and the initial activity overlapped each time the influence of Pt abrasion seems to be negligible. Interestingly, although comparable amounts of Fe and Pt particle were found while performing the electrochemistry in K^+ containing electrolytes, no significant increase in the activity for the side reactions was found.

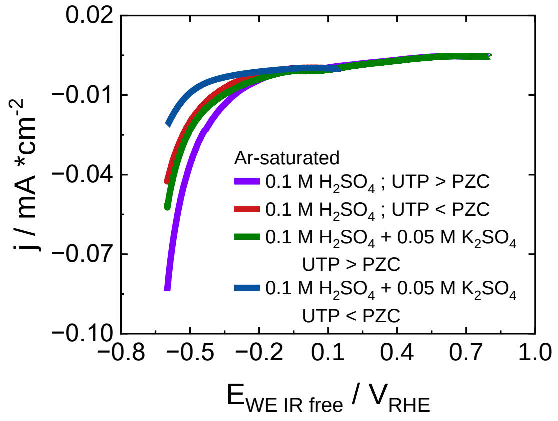

Figure S8: HER measurements: Anodic voltammetric scans in 0.1 M H_2SO_4 + 0.05 M K_2SO_4 and 0.1 M H_2SO_4 with UTP below and above PZC. Conditions: 1600 rpm, 20 mV/s and Ar -saturated electrolytes.

Supporting Note 5: *In situ* XPS measurements

In situ XPS measurements were carried out at the ISISS beamline NAP-XPS end station in BESSY II. For all measurements the *in situ* membrane-electrode flow cell developed by the Electronic Structure group at the Fritz-Haber Institute[3], was used. The flow cell allowed the combination of in situ liquid phase electrochemical experiments with XPS measurements. The flow cell utilizes a proton exchange membrane (PEM, Nafion N117) in the combination with a transparent 2D working electrode (trilayer graphene TLG). The graphene layer was directly used as the catalyst. Utilizing the liquid confinement effect, the catalyst is in constant contact with the electrolyte. The main body of the cell is made of polyether ether ketone (PEEK). A Pt wire is used as the counter electrode and an Ag/AgCl electrode as the reference electrode. The TLG is used as the catalyst, current collector, transparent electrode for the photoelectrons and as an evaporation barrier for the liquid electrolyte, enabling the formation of a confined thin-film of liquid environment. The electrolytes were pumped through the flow cell using a peristaltic pump.

Working electrode fabrication

The polymer support of the TLG was removed by carefully placing the samples under MilliQ water, resulting in a floating graphene layer and a sacrificial polymer layer on top. The TLG was afterwards transferred onto the PEM. The TLG on the PEM is stabilized by Van der Waals interaction. The sacrificial layer on the TLG was removed by washing in ethyl acetate. The resulting TLG on PEM was rinsed with MilliQ water, dried at room temperature for 24 h and stored under N_2.

Survey and high-resolution spectra were acquired at pass energies of 50 eV and 20 eV and an excitation energy of 1100 eV and 1000 eV respectively. Data analysis and quantification were performed using CasaXPS software. A Shirley background subtraction was applied to the C 1s – K 2p spectra. Fitting of the C 1s – K 2p spectra was performed using a model which includes 10 singlets corresponding to different C 1s species and one doublet corresponding to the K^+ species. The different C species are C^{sp2}, C^{sp3}, C-O(H), C=O, O-C=O, and C1s satellites, which account for the graphene and adventitious carbon with different functional groups, and CF2, CFO, CF2O, and CF3, which account for the Nafion membrane. The K /(C + K) molar ratios were calculated based on the integrated K 2p peak area, the integrated area of the whole C 1s-K 2p region and the corresponding cross sections at 1000 eV.

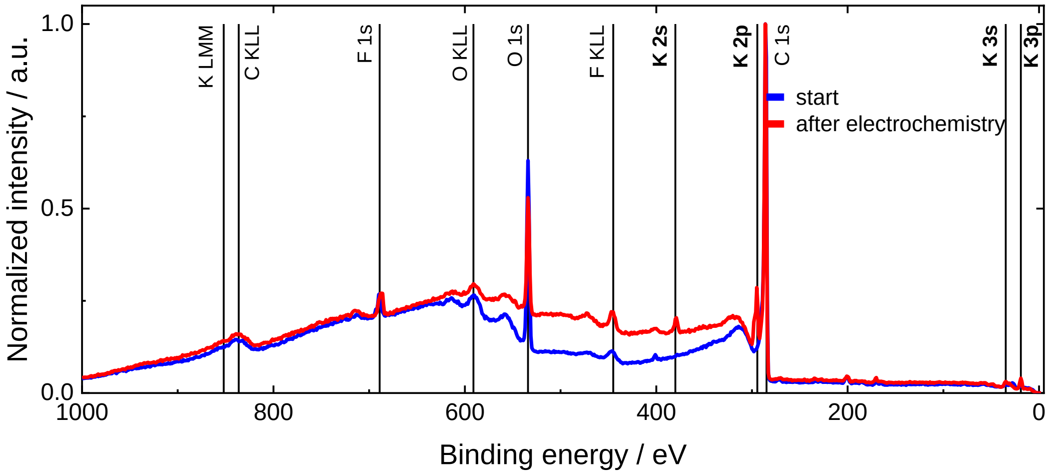

Figure S9: XPS survey spectra of trilayer graphene on Nafion N117 with identification of the observed core levels. Survey spectra were acquired at pass energy of 50 eV and an excitation energy of 1100 eV.

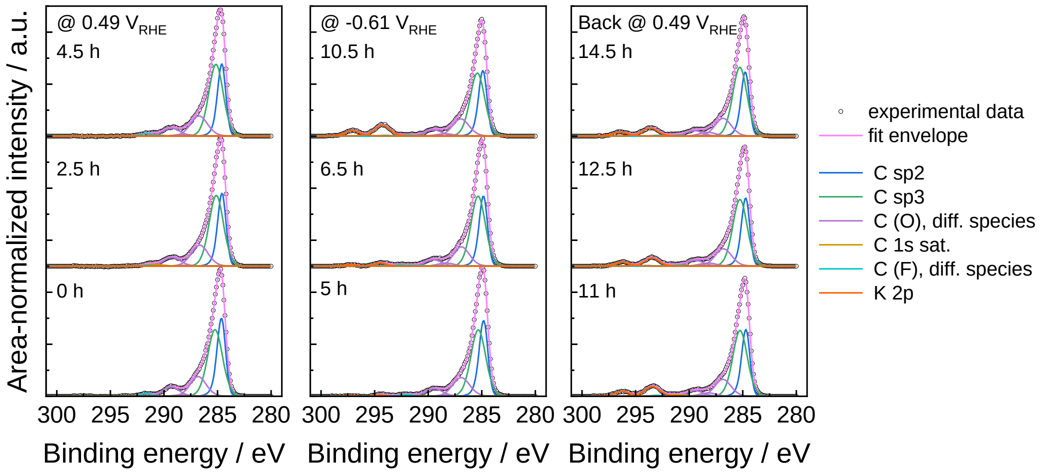

Figure S10: XPS spectra of trilayer graphene on Nafion N117 (Spot No.2) of K 2p and C 1s region. High-resolution spectra were acquired at pass energies of 20 eV and an excitation energy of 1000 eV.

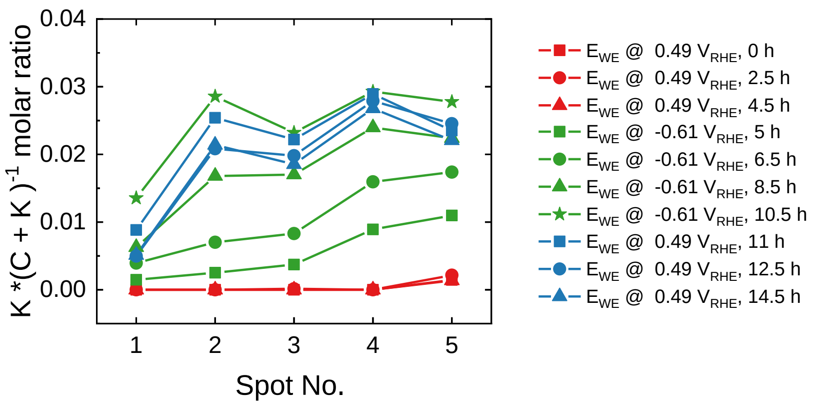

Figure S11: Fitted K/(C + K) molar ratios of trilayer graphene on Nafion N117 for all spots. High-resolution spectra were acquired at pass energy of 20 eV and an excitation energy of 1000 eV.

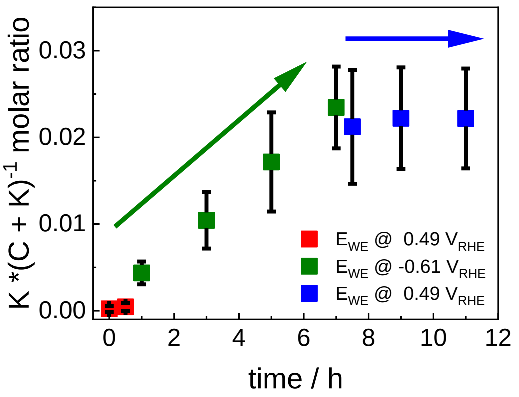

Figure S12: Averaged K/(C + K) molar ratios of whole sample over time as function of applied potential for short electrochemical protocol.

Supporting Note 6: Computational Details

The density functional theory (DFT) calculations were carried out with the Quantum ESPRESSO[4] simulation package, utilizing GGA-PBE functionals to describe the exchange-correlation energy[5] and SSSP pseudopotentials to account for the core electrons[6]. A 20 Å vacuum was set along the surface perpendicular direction to avoid interactions between periodic images and the Brillouin zone was sampled with a Monkhorst–Pack *k*-point grid of 2x2x1 was employed for the supercell ORR calculations. In this work, the system was modelled by taking graphene sheets with 8x8 rings and introducing defects in the network, as shown in Figure S13. The defects were chosen according to the following criteria: with experimental evidence of being found in carbon-based materials, and previous theoretical calculations indicating their catalytic activity towards the $2e^-$ ORR.[7] The computational hydrogen electrode (CHE) framework was employed to describe the ORR thermodynamics.[8] In this model, the free energy of an electron-proton pair is equivalent to half of an H_2 molecule considering a system in equilibrium at pH = 0 and the ORR intermediate species adsorption energies (*OOH, *OH, and *O) are calculated considering gaseous H_2 and H_2O molecules as references.[8] Since the *OOH intermediate species is key for the H_2O_2 electrogeneration, ΔG_{*OOH} was used as the catalytic activity descriptor.[9] Finally, the field effect was modelled by applying a saw-tooth potential corresponding to a range from -0.6 to 0.6 V/Å, allowing the adsorbates to relax at each applied potential value. [10-12] The Gibbs free energy was then calculated for each applied field with respect to the system without any field effects (E = 0 V/Å), treating vibrational and entropic contributions as constant through the selected potential range.[10, 11] The limiting potential (U_L) is defined by the lowest potential value in which ΔG is still negative for all reaction steps.

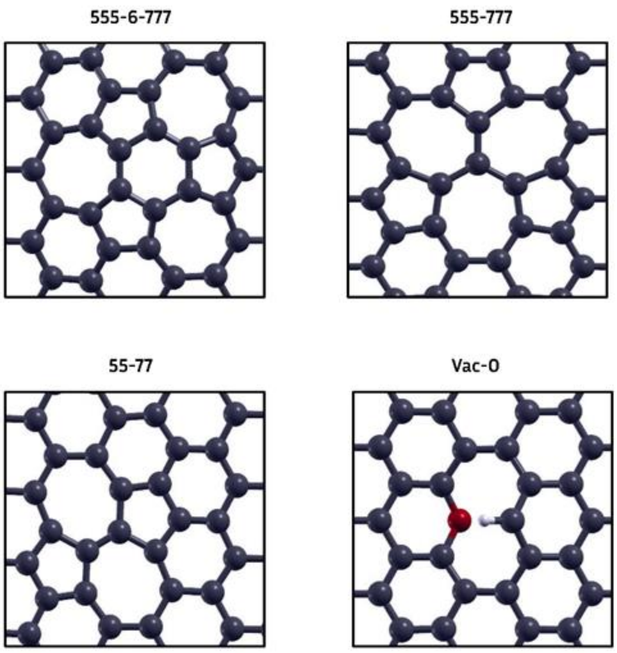

Figure S13: Typical defects and functional groups in carbon-based materials used for the computational calculations.

Table S1: Coordinates of the optimized surface models.

```
CRYSTAL
 9.83804857654350 17.03999203700218 0.00000000000000
 -9.83804857654350 17.03999203700218 0.00000000000000
 0.00000000000000 0.00000000000000 14.99999396026727

  6    0.00164469249547    1.44535070668984    6.74607592819920
  6    0.00159690417451    2.87018972744504    6.71478568729818
  6   -1.23384089968706    3.58371648372522    6.73404792204226
  6   -1.23443700475880    5.00620451893107    6.80066383871943
  6   -2.46796939406436    5.71422225430567    6.90911636305113
  6   -2.47053481378523    7.12328868014360    7.08768870364915
  6   -3.68889829869070    7.84350048430777    7.24348719091705
  6   -3.68506492273895    9.25277516100038    7.49967340026392
  6   -4.90506794795385    9.97611858512511    7.56979527152942
  6   -4.89118914621580   11.40303374147011    7.74777229836715
  6   -6.13444994396554   12.10688241616097    7.58880232037625
  6   -6.15280929800485   13.52909786772620    7.53665689037256
  6   -7.37482762582695   14.22887316980859    7.28583582786541
  6   -7.39328758276720   15.64634552425433    7.13917375891875
  6   -8.60941551575297   16.35221092119475    6.94685659535506
  6   -8.60611159111004   17.77367731175002    6.83306891217156
  6    1.23518909897736    3.58232071582548    6.75422553291777
  6    1.23797031727756    5.00489584583862    6.81162515180587
  6    0.00189756772847    5.71511476819259    6.84779761474106
  6    0.00424813627408    7.12257138508680    7.03949328805499
  6   -1.23933245314747    7.81570260342606    7.18262836192179
  6   -1.24531516921544    9.18369075965237    7.53077210174206
```

6	-2.45809930886643	9.93341662579226	7.71985593260764
6	-2.45363958273020	11.31606167152922	8.14686005267486
6	-3.67725173935282	12.09817887386824	8.07419619843289
6	-2.71202592821674	14.43471884495037	8.61917440549795
6	-4.95397068862399	14.25445664067712	7.76827418961208
6	-5.00573232784209	15.67825396071112	7.60708583851441
6	-6.18607193306790	16.36915721883554	7.25295249310585
6	-6.16141018344255	17.78893864385822	7.10985835872256
6	-7.37707258980773	18.49026068476180	6.89292126857207
6	-7.37104269931302	19.91293940599854	6.83842814751367
6	2.47289034661015	5.70863652446795	6.92187316041462
6	2.47843617855216	7.11929975943967	7.07878598973382
6	1.24828116180879	7.80904876258348	7.16995657252408
6	1.26102263790875	9.18251499508982	7.50654065849883
6	0.00965330871657	9.79264060548262	7.73454424469341
6	0.02189473451587	11.04996443338374	8.28064037730834
6	-1.16889096676539	11.79791928714323	8.55605499791292
6	-1.39650525789042	14.22483400328014	9.14661214162567
6	-3.74692069395423	13.56828175030308	8.18904713068827
6	-2.76859282059415	15.83107443016024	8.37635796226775
6	-3.85919240986448	16.46011397845032	7.83541496207791
6	-3.75534845236450	17.84405097763954	7.60690974758531
6	-4.94450929957525	18.52038133296605	7.25359297934796
6	-4.92177951838283	19.92702225907345	7.14262634652856
6	-6.14179907447075	20.62663406754425	6.95340469121848
6	-6.13480586390314	22.04380230073211	6.97388540997194
6	3.69993808064175	7.83752284828519	7.22515671079780
6	3.70030829231251	9.24653873521074	7.46183223750063
6	2.47381853107638	9.92367809312719	7.67600649226358
6	2.47051890369307	11.30995883927715	8.10435486378953
6	1.20408827375711	11.77201224216192	8.51782400780659
6	0.73022910345228	13.02659377813653	9.03189988431444
6	-0.71128637280848	13.01981019309059	9.07303285275229
6	-0.69130329902566	15.44403925482654	9.14111188684034
6	-1.53280047904080	16.48065617278670	8.61525031407798
6	-1.29927822035707	17.80916923467621	8.18874179231122
6	-2.49787087065308	18.51227239778746	7.77129607789532
6	-2.46848826947097	19.91313051802923	7.54435990327095
6	-3.68553019258647	20.62056804849097	7.29303978646474
6	-3.67971361951525	22.03873086341405	7.24099770041944
6	-4.90710454304958	22.75009991707055	7.11947096985205
6	-4.90784119940231	24.17009503040936	7.20418157274341
6	4.92254028484121	9.97001458733758	7.52800897235463
6	4.90893833562815	11.39692968574661	7.69057251689858
6	3.70281088231995	12.09309821791449	8.00931883055569
6	3.76308671999452	13.55111793375595	8.08967382820083
6	2.72754682717304	14.41700867002656	8.49954802416539
6	2.76142747467439	15.80176739556778	8.25575615382795
6	1.52035912638593	16.45954834102665	8.53357254496545
6	1.29695951036417	17.81413207101302	8.13196949867053
6	0.00929523719775	18.48461156104171	8.07173119992541
6	0.00468045062649	19.88387184804617	7.75130284844557
6	-1.22712426224386	20.60853020746040	7.59888758431543
6	-1.22367437637704	22.02637668685931	7.46078932542056
6	-2.45093475687720	22.74301816283593	7.34720982265323
6	-2.45195091612521	24.16662308943184	7.33956314223216
6	-3.68260351086763	24.88066804890986	7.31333593029252

6	-3.68762766454299	26.30316951503742	7.40743451490382
6	6.15076271335460	12.09863217344040	7.51892470451240
6	6.16516282246486	13.51826101726239	7.44660844363047
6	4.96410989855975	14.24141692138674	7.65675985451322
6	4.99959844425490	15.66247505077280	7.48265423611667
6	3.84440633344865	16.44075029893121	7.70824494278280
6	3.74577343090328	17.83287479959031	7.50496032963515
6	2.49213291637945	18.50954492383804	7.70268209152267
6	2.46902492124755	19.91331455675923	7.48943385088687
6	1.23347732082865	20.60917885690928	7.56895851936634
6	1.23266987479739	22.02894015940537	7.44165822662367
6	0.00563696670500	22.74401701138516	7.44481115485415
6	0.00634422204956	24.16493563265242	7.39739823344492
6	-1.22302885675491	24.87678460234864	7.38101195204284
6	-1.22429358905727	26.29909767432425	7.37957712012057
6	-2.45327950523327	27.00783915268864	7.40412958423455
6	-2.46063257686075	28.42891876439266	7.39895903381646
6	7.38564120251088	14.22281365832828	7.20846424351900
6	7.39649462919438	15.63943643114703	7.05851486289597
6	6.18320934426792	16.36007940504571	7.14566655580443
6	6.15642678880245	17.78133226746077	7.01488379996398
6	4.93426232660679	18.51215963564015	7.15776211143416
6	4.91921301155760	19.92542520967577	7.07011908922354
6	3.68751772435015	20.62137970420768	7.23210019300201
6	3.68615035365459	22.04148420236738	7.20155785779985
6	2.45984305378638	22.74629628412803	7.32313078534863
6	2.46349991055155	24.17058667166761	7.33581273124226
6	1.23672728672654	24.87884071253979	7.38332102111309
6	1.23979465549145	26.30107782534290	7.39267159734809
6	0.00830992318344	27.00911375090901	7.37467994609241
6	0.00848111605053	28.42781050887056	7.32774475349082
6	-1.22609706667296	29.13552153631650	7.30004418064443
6	-1.22376393410084	30.55144822034855	7.18905873783261
6	8.61610254755659	16.34970134955150	6.90374167471524
6	8.60862847875348	17.77136952428448	6.79648442340227
6	7.37549972982394	18.48711613144729	6.83005278238600
6	7.37160446270860	19.91285570044566	6.79092471014088
6	6.14203062376586	20.62777679156224	6.89922678453316
6	6.13905216931638	22.04732788019656	6.94119912263304
6	4.91424213091528	22.75508310086184	7.09302929249877
6	4.91857654884298	24.17545740003146	7.20436016867150
6	3.69514432717742	24.88574480440942	7.32345895521649
6	3.70283831670221	26.30722242261546	7.43964184493557
6	2.47010481924468	27.01230773383242	7.43859415385742
6	2.47665592069873	28.43549447687175	7.44960477792400
6	1.24202092029330	29.13906071199060	7.33342051870548
6	1.23744042128893	30.55496865543940	7.22793980067719
6	0.00674064916836	31.26117559346022	7.10555439695555
6	0.00423780829069	32.67740433520711	6.96108141362745
6	0.69216712986473	15.45383891164704	9.05716638714091
6	1.40871115062588	14.20074290854621	9.02035583796264

CRYSTAL
19.73449132885161 0.00000000000000 0.00000000000000
-9.86724566442581 17.09056285306872 0.00000000000000
0.00000000000000 0.00000000000000 15.00000924863815

6	0.01738318180000	0.03954680970000	2.49988614530000
6	0.02339719000000	1.46876272420000	2.49981924270000
6	-1.22235522400000	2.15934978320000	2.49992811410000
6	-1.22474107580000	3.58373740470000	2.49977490090000
6	-2.47071187430000	4.28377594350000	2.49997804230000
6	-2.48146904730000	5.70848015670000	2.49991840810000
6	-3.72991673590000	6.41526883390000	2.49982202450000
6	-3.75504837310000	7.83953089150000	2.49982024160000
6	-5.00532700640000	8.54829122890000	2.49980051910000
6	-5.00479949450000	9.97263904410000	2.49971496470000
6	-6.25104494840000	10.67914461380000	2.49996231080000
6	-6.20673678130000	12.10676506220000	2.49965127510000
6	-7.43214886740000	12.82106144740000	2.49970061090000
6	-7.40943230850000	14.25535213360000	2.49969519020000
6	-8.62731080990000	14.98715925660000	2.49953165800000
6	-8.61718307720000	16.41833065280000	2.49980264900000
6	2.48475034300000	0.04825301340000	2.49981303970000
6	2.49594858660000	1.48546881770000	2.49979237830000
6	1.26221033990000	2.19730117540000	2.49970500290000
6	1.27348275670000	3.62982878970000	2.49996386830000
6	0.01492636380000	4.30540752990000	2.49965625700000
6	0.00917715210000	5.72373811720000	2.49970997670000
6	-1.24454849950000	6.42106332150000	2.49970009330000
6	-1.26930343740000	7.84172227330000	2.49953423460000
6	-2.51378419020000	8.54860787640000	2.49981072480000
6	-2.51381086810000	9.97205409200000	2.49989191800000
6	-3.75461308410000	10.68152972990000	2.49982708560000
6	-3.72978588000000	12.10558593580000	2.49993185970000
6	-4.96215469030000	12.81961920820000	2.49977562490000
6	-4.94547370440000	14.24868588670000	2.49997988800000
6	-6.17388803570000	14.97019580090000	2.49991907250000
6	-6.16163206420000	16.40479240230000	2.49981830180000
6	4.94066287810000	0.02450745440000	2.49965901930000
6	4.94576484060000	1.45882105950000	2.49979661540000
6	3.73164519030000	2.21110954520000	2.50010775200000
6	3.75153420440000	3.66010695360000	2.50119104260000
6	2.51890297860000	4.37148605600000	2.50103312340000
6	2.53845679460000	5.81241095110000	2.50157022000000
6	1.24944323540000	6.44076992290000	2.50034200650000
6	1.20660453010000	7.84876226270000	2.50005810350000
6	-0.03638929490000	8.55138056790000	2.49927917970000
6	-0.03658338530000	9.96812293800000	2.49925307990000
6	-1.26920358650000	10.67829859040000	2.49968158340000
6	-1.24460355500000	12.09895373440000	2.49981974020000
6	-2.48125801670000	12.81166674770000	2.50002135830000
6	-2.47030967370000	14.23633744070000	2.50005320080000
6	-3.70425843310000	14.95696950600000	2.50005254240000
6	-3.69739604160000	16.38613294490000	2.49980991210000
6	7.40904957530000	-0.00150409240000	2.49954491980000
6	7.40805320170000	1.42595873040000	2.49924416170000
6	6.17742161550000	2.16333866480000	2.49996825410000
6	6.17853286880000	3.59580329560000	2.50161630860000
6	4.98981479120000	4.39810176910000	2.50264299150000
6	5.03988694580000	5.87763134410000	2.50415955810000
6	3.79515228740000	6.59636668470000	2.50313805300000
6	3.74336893350000	8.05421931130000	2.50302562040000
6	2.41774138180000	8.56125438830000	2.50113182850000

6	2.41783336900000	9.95815652240000	2.50064620740000
6	1.20658529720000	10.67070401570000	2.49955008260000
6	1.24935726190000	12.07918796080000	2.49970621050000
6	0.00932132830000	12.79597066550000	2.49965759700000
6	0.01517302500000	14.21475279340000	2.49988784460000
6	-1.22441020440000	14.93592650750000	2.50007522750000
6	-1.22220523070000	16.36064614290000	2.49991114550000
6	9.89066848410000	-0.03912985860000	2.49972076630000
6	9.88466629020000	1.39030018730000	2.49906582980000
6	8.65070769650000	2.11496739920000	2.49909096430000
6	8.64925620310000	3.53759501600000	2.50040656000000
6	7.41911794540000	4.26275991430000	2.50248773380000
6	7.43075820330000	5.66775855140000	2.50498062020000
6	6.32816298300000	6.56295450430000	2.50529516060000
6	6.90626927060000	7.97207765590000	2.50604102170000
6	4.67488811050000	9.26141992030000	2.50376908360000
6	6.16237059930000	9.26001291980000	2.50448879580000
6	3.74437337890000	10.46505817160000	2.50212963790000
6	3.79532973090000	11.92478809070000	2.50146371390000
6	2.53830891640000	12.70790018750000	2.50035492620000
6	2.51858865830000	14.14926854940000	2.50000588000000
6	1.27323384270000	14.89063513970000	2.49988092420000
6	1.26233449350000	16.32328386770000	2.49993735400000
6	12.36322301430000	-0.05512722280000	2.49993859740000
6	12.35186719250000	1.38187611310000	2.49972105390000
6	11.11704262910000	2.10203214930000	2.49906571590000
6	11.10686393970000	3.53316369580000	2.49909439610000
6	9.87578409590000	4.24573763630000	2.50040796150000
6	9.86254673830000	5.67370354630000	2.50247716110000
6	8.64009096680000	6.36615202950000	2.50497376610000
6	8.41588275380000	7.76847348520000	2.50529117920000
6	6.90486405250000	10.54901029920000	2.50377895610000
6	6.32826124710000	11.95669461270000	2.50214446780000
6	5.03872588080000	12.64263514780000	2.50147391150000
6	4.98916085420000	14.12273647420000	2.50037314050000
6	3.75096537220000	14.86066175960000	2.50001101860000
6	3.73154348040000	16.30983709890000	2.49988542810000
6	14.81298652920000	-0.02896636090000	2.49989143990000
6	14.80797101670000	1.40505862860000	2.50007231200000
6	13.57285103450000	2.11527436400000	2.49991272810000
6	13.56042875010000	3.54979162730000	2.49955124180000
6	12.32488762780000	4.26459653350000	2.49924556720000
6	12.30159891660000	5.69904251340000	2.49996401450000
6	11.06061696020000	6.41436115620000	2.50159755720000
6	10.95988987630000	7.84494731450000	2.50260344490000
6	9.65347925060000	8.54117404700000	2.50412503310000
6	9.65340383970000	9.97885923740000	2.50311692610000
6	8.41663900680000	10.75225992380000	2.50302722500000
6	8.64048358580000	12.15377515980000	2.50114925620000
6	7.43065845620000	12.85235955250000	2.50067958750000
6	7.41923295150000	14.25733084000000	2.49958388830000
6	6.17795604880000	14.92471820990000	2.49972883740000
6	6.17736673600000	16.35713831080000	2.49966652660000
6	17.27514569420000	0.00374330260000	2.49982218330000
6	17.27626104040000	1.43100283740000	2.50001623150000
6	16.03697189090000	2.13408948020000	2.50004705620000
6	16.02969362470000	3.56299521470000	2.50005319250000

6	14.78859606920000	4.27162014510000	2.49981907320000
6	14.77199544250000	5.70065858590000	2.49966653170000
6	13.52744259460000	6.41336146350000	2.49979933600000
6	13.48292519450000	7.84097254670000	2.50010716940000
6	12.21813700820000	8.54834143890000	2.50116946860000
6	12.21839126960000	9.97141633960000	2.50101610820000
6	10.96073295470000	10.67506486750000	2.50154988980000
6	11.06113798490000	12.10548460240000	2.50033200550000
6	9.86318009670000	12.84657370000000	2.50006805540000
6	9.87613157170000	14.27430832530000	2.49929232650000
6	8.64912085230000	14.98268252230000	2.49927167640000
6	8.65069562100000	16.40524330200000	2.49968514030000

CRYSTAL
19.73449132885161 0.00000000000000 0.00000000000000
-9.86724566442581 17.09056285306872 0.00000000000000
0.00000000000000 0.00000000000000 15.00000924863815

6	-0.00199488180000	0.03295676020000	2.50021491880000
6	-0.00490664640000	1.46133525680000	2.50010065170000
6	-1.26181955030000	2.12793620200000	2.50079547020000
6	-1.28798021150000	3.54355632710000	2.50176542120000
6	-2.52169892370000	4.25183668120000	2.50193105890000
6	-2.56352106010000	5.67847403970000	2.50378461610000
6	-3.76635056740000	6.40619011170000	2.50107809990000
6	-3.76645272420000	7.83609024530000	2.50108763330000
6	-4.96488338370000	8.56670586440000	2.49933062640000
6	-4.95355943960000	9.99634013280000	2.49929166670000
6	-6.16714401680000	10.72121176880000	2.49998783410000
6	-6.16709790120000	12.14982536090000	2.49999409370000
6	-7.39200094020000	12.86743556080000	2.50033454690000
6	-7.39460045100000	14.29607297970000	2.50004689880000
6	-8.62699730560000	15.00423896430000	2.50015481980000
6	-8.62644564490000	16.43469620820000	2.50006360250000
6	2.47006501550000	0.08438786900000	2.49969974830000
6	2.47087721670000	1.52357295660000	2.49908113920000
6	1.22633352570000	2.21505268580000	2.49891688620000
6	1.21014859050000	3.65170056790000	2.49732854540000
6	-0.07643662080000	4.27520693710000	2.49973663630000
6	-0.17992997400000	5.67844373500000	2.49843492610000
6	-1.37661431330000	6.40833226680000	2.50449279940000
6	-1.37672217910000	7.83392387550000	2.50449985970000
6	-2.56364285600000	8.56380045750000	2.50381366460000
6	-2.52175076060000	9.99045751800000	2.50196256740000
6	-3.73263788750000	10.71585725880000	2.49980098790000
6	-3.71988503070000	12.14205086160000	2.49956319060000
6	-4.94218580930000	12.86752892920000	2.49966080330000
6	-4.93936638200000	14.29611491400000	2.49994472230000
6	-6.16696108290000	15.02080074890000	2.49999448440000
6	-6.16695480900000	16.45318089330000	2.49999520030000
6	4.93044871980000	0.08439047770000	2.50030632350000
6	4.92958814490000	1.52354388370000	2.50100204960000
6	3.70020223440000	2.27791736560000	2.50007113850000
6	3.70019358980000	3.73363202730000	2.50008249690000
6	2.43566608490000	4.44867625060000	2.49500424060000
6	2.25355896680000	5.88695667440000	2.48795675340000
6	0.97385507190000	6.41388087850000	2.48817050900000

6	0.97389748340000	7.82827125350000	2.48812187930000
6	-0.17995354310000	8.56374203040000	2.49837087660000
6	-0.07647657200000	9.96705603810000	2.49966432050000
6	-1.28807015680000	10.69873569110000	2.50177134540000
6	-1.26202338930000	12.11433978640000	2.50081582730000
6	-2.48595364480000	12.84585289960000	2.50016926040000
6	-2.47910749280000	14.26963591140000	2.49994796360000
6	-3.70700246660000	15.00427951370000	2.49982611390000
6	-3.70743513980000	16.43470839990000	2.49991179610000
6	7.40259402060000	0.03293732070000	2.49976132390000
6	7.40549943090000	1.46135198890000	2.49989212470000
6	6.17413228440000	2.21499447710000	2.50115621970000
6	6.19029600040000	3.65166187470000	2.50278523840000
6	4.96462920350000	4.44869314410000	2.50512705120000
6	5.14677865800000	5.88683020590000	2.51210856580000
6	4.36004537490000	7.12109390740000	2.50954356280000
6	3.04023853430000	7.12110111270000	2.49047586750000
6	2.25347131370000	8.35528946440000	2.48787149440000
6	2.43571714960000	9.79337585510000	2.49486109510000
6	1.21011107330000	10.59042056300000	2.49720239460000
6	1.22625052600000	12.02713092260000	2.49883779750000
6	-0.00507101900000	12.78081595670000	2.50010194440000
6	-0.00210644720000	14.20921424520000	2.50023532950000
6	-1.23372951290000	14.95279310690000	2.50012999810000
6	-1.23301174270000	16.38001643900000	2.49986725790000
6	9.87965200790000	-0.02746014410000	2.50005099240000
6	9.88647020830000	1.39634254180000	2.49982933060000
6	8.66245441970000	2.12786144310000	2.49917896760000
6	8.68850523180000	3.54343857950000	2.49822572750000
6	7.47689674790000	4.27504647670000	2.50032542380000
6	7.58027150150000	5.67836463620000	2.50162675800000
6	6.42641178100000	6.41377158350000	2.51187011860000
6	6.42647043530000	7.82817016750000	2.51185772510000
6	5.14678501750000	8.35518625010000	2.51208095210000
6	4.96468830460000	9.79341709910000	2.50502994500000
6	3.70019475720000	10.50843867930000	2.49992888460000
6	3.70017430210000	11.96421576370000	2.49993270130000
6	2.47077214000000	12.71858209090000	2.49899796120000
6	2.46998659900000	14.15778260690000	2.49969224410000
6	1.24069683360000	14.89801761820000	2.50008612070000
6	1.24031585760000	16.32840821130000	2.50017287050000
6	12.33996086190000	-0.05401723430000	2.50005238570000
6	12.34275901020000	1.37455003450000	2.50033514610000
6	11.12042749020000	2.10005885900000	2.50043275410000
6	11.13310687120000	3.52628753340000	2.50019307700000
6	9.92221825990000	4.25169966830000	2.49803402120000
6	9.96404446880000	5.67833271340000	2.49619082230000
6	8.77710084540000	6.40814448670000	2.49551327500000
6	8.77703207150000	7.83371519720000	2.49551108430000
6	7.58034146750000	8.56358341430000	2.50157073650000
6	7.47686205420000	9.96686319600000	2.50025988400000
6	6.19026454300000	10.59037382980000	2.50267675400000
6	6.17402989970000	12.02707133920000	2.50108016310000
6	4.92947729890000	12.71854889710000	2.50091910820000
6	4.93036636840000	14.15778178060000	2.50029914560000
6	3.70019095520000	14.87954685050000	2.50000471250000
6	3.70029044480000	16.31189360130000	2.50000492220000

```
 6   14.79521857550000   -0.05400162870000    2.49995279720000
 6   14.79257871770000    1.37463037090000    2.49966595570000
 6   13.56766551880000    2.09225552340000    2.50000494330000
 6   13.56762353930000    3.52093459190000    2.50001046590000
 6   12.35400677260000    4.24579383990000    2.50070400760000
 6   12.36526385350000    5.67541940250000    2.50065964030000
 6   11.16685776730000    6.40603165740000    2.49890865170000
 6   11.16673842270000    7.83596730580000    2.49891589790000
 6    9.96391095250000    8.56367926120000    2.49621113080000
 6    9.92208414840000    9.99038274350000    2.49807007750000
 6    8.68834604590000   10.69865474110000    2.49823471270000
 6    8.66220784230000   12.11429259330000    2.49920680960000
 6    7.40529955990000   12.78080264400000    2.49989542640000
 6    7.40249230950000   14.20914937640000    2.49978157330000
 6    6.15972991120000   14.89798411800000    2.49993278360000
 6    6.16034915110000   16.32839715000000    2.49984362340000
 6   17.25555246600000   -0.02751351710000    2.49994693760000
 6   17.24876807060000    1.39634164490000    2.50014953170000
 6   16.01479983490000    2.10016380980000    2.49955736900000
 6   16.00196462710000    3.52638000660000    2.49978360400000
 6   14.78103887290000    4.24596115710000    2.49929906790000
 6   14.76969749490000    5.67559141910000    2.49933107870000
 6   13.56737215360000    6.40513572590000    2.49999407210000
 6   13.56739764220000    7.83709812840000    2.49999273480000
 6   12.36510298970000    8.56669640250000    2.50066017500000
 6   12.35381012200000    9.99632886060000    2.50069739170000
 6   11.13289467480000   10.71588021620000    2.50021766220000
 6   11.12016337090000   12.14203241540000    2.50044745290000
 6    9.88620725670000   12.84583911590000    2.49985477470000
 6    9.87949425710000   14.26963356590000    2.50005408990000
 6    8.63410713920000   14.95269060690000    2.49987747900000
 6    8.63367833990000   16.37997813610000    2.50012228680000

CRYSTAL
 19.73449132885161 0.00000000000000 0.00000000000000
 -9.86724566442581 17.09056285306872 0.00000000000000
 0.00000000000000 0.00000000000000 15.00000924863815

 6   -0.00186586340000    0.00648454320000    2.48403312480000
 6    0.00516425100000    1.43544844000000    2.48368775460000
 6   -1.22821974800000    2.14316712400000    2.47738087820000
 6   -1.22487622400000    3.56956326910000    2.47019326010000
 6   -2.45911268360000    4.27789310210000    2.46051249400000
 6   -2.46084007700000    5.70202354810000    2.44457700800000
 6   -3.69564897940000    6.41304026300000    2.44505171520000
 6   -3.70370232300000    7.83743725800000    2.43271780890000
 6   -4.93551980460000    8.54966910100000    2.44871778720000
 6   -4.94202224000000    9.97528587770000    2.44285154300000
 6   -6.17551552130000   10.68683543130000    2.47593184480000
 6   -6.17713285190000   12.11137554260000    2.47373108010000
 6   -7.41342277940000   12.82062610020000    2.49830717370000
 6   -7.41033847070000   14.24813114040000    2.48838451250000
 6   -8.64356259040000   14.95747578850000    2.49500533910000
 6   -8.63709721210000   16.38601597060000    2.49028247740000
 6    2.46432169860000    0.01473281530000    2.49582316600000
 6    2.47179555680000    1.44506515830000    2.50605045360000
 6    1.24110753300000    2.15768759900000    2.49859263950000
```

6	1.24862636620000	3.58714187320000	2.50201787680000
6	0.00910593520000	4.28722960080000	2.47201108650000
6	0.00840078730000	5.70947110990000	2.43959513160000
6	-1.22894383010000	6.41685882610000	2.42557797680000
6	-1.24255176470000	7.84225082190000	2.39794909880000
6	-2.47438556050000	8.55314455650000	2.40565969080000
6	-2.47944672680000	9.97774561230000	2.38999457010000
6	-3.71155857500000	10.69016314210000	2.41289672990000
6	-3.71218957430000	12.11462192730000	2.41534732750000
6	-4.94476729260000	12.82650095290000	2.44569969960000
6	-4.94313751030000	14.25228040050000	2.45492034750000
6	-6.17598614210000	14.96456677740000	2.47634943410000
6	-6.17204061990000	16.39188056760000	2.48995241460000
6	4.92935121720000	0.01865546270000	2.49984913700000
6	4.93432035270000	1.44349850240000	2.53454370450000
6	3.70517913130000	2.16576117500000	2.52976453030000
6	3.71236515640000	3.59587237850000	2.55755043110000
6	2.48422424500000	4.31376408540000	2.53192383430000
6	2.49444873970000	5.74877631380000	2.49149223220000
6	1.24360601290000	6.43080829650000	2.42315988770000
6	1.21455066120000	7.84891436840000	2.36125524130000
6	-0.01673415000000	8.56034349180000	2.35821890910000
6	-0.02249840390000	9.98257357460000	2.32157098090000
6	-1.25035126480000	10.69339600830000	2.35389522830000
6	-1.24943159010000	12.12002620880000	2.35969539370000
6	-2.47910406100000	12.82998039140000	2.39503735330000
6	-2.47736534400000	14.25591681860000	2.41726667800000
6	-3.70980878030000	14.96771717920000	2.44690457900000
6	-3.70623044300000	16.39260980200000	2.47526592910000
6	7.39537917740000	0.01318487490000	2.47618429510000
6	7.40047420810000	1.43743651490000	2.52117738440000
6	6.16929814840000	2.15638398570000	2.56792876000000
6	6.17682225930000	3.57812909140000	2.64353453390000
6	4.94797359450000	4.31740136480000	2.65000828390000
6	4.96582275060000	5.74126552210000	2.74505553720000
6	3.74499020160000	6.48431579160000	2.50668507180000
6	3.65756861200000	7.88977081840000	2.24224202960000
6	2.43336736070000	8.57009403230000	2.26015325920000
6	2.42702522620000	9.99759578680000	2.21649353290000
6	1.20984342850000	10.70034897280000	2.27602635760000
6	1.21501772630000	12.12641547920000	2.32004950560000
6	-0.01479176870000	12.83421030670000	2.35300338010000
6	-0.01084386210000	14.25643943680000	2.39005798850000
6	-1.24380782670000	14.96780533270000	2.41513615540000
6	-1.23930928450000	16.39063486050000	2.44386515660000
6	9.86620541220000	0.00628984660000	2.44811790290000
6	9.87209535490000	1.42709364540000	2.47800397460000
6	8.63981454510000	2.13924639180000	2.51957741110000
6	8.64848422510000	3.55856310580000	2.57489517670000
6	7.41972344260000	4.27059955290000	2.65186432590000
6	7.43992163890000	5.68271166660000	2.78624654920000
6	6.22699281810000	6.39398880600000	3.00755462730000
6	6.33007365470000	7.74407791190000	3.38426753730000
6	4.86790903360000	9.98819243260000	2.24164539640000
6	3.66599010260000	10.70689569360000	2.25933140890000
6	3.68151110210000	12.12253255190000	2.36112437930000
6	2.44965362400000	12.83250344580000	2.35738234600000

6	2.45759853060000	14.25270468910000	2.39775666100000
6	1.22572905810000	14.96352114130000	2.40531517530000
6	1.23151960550000	16.38563113260000	2.43306576460000
6	12.33578679160000	0.00107212750000	2.44628184900000
6	12.34001827640000	1.42385436870000	2.46147650500000
6	11.10802560320000	2.13475952050000	2.47870159740000
6	11.11413721000000	3.55719927040000	2.51266484030000
6	9.88491286750000	4.26670854370000	2.57398016910000
6	9.89381945650000	5.68594384670000	2.63521766120000
6	8.67024452840000	6.39232686100000	2.75781496110000
6	8.67009673230000	7.81292822580000	2.78438401870000
6	7.44731820950000	8.50803483730000	3.00578501510000
6	7.38264148770000	9.92697338010000	2.74309882070000
6	6.12875532410000	10.61436603300000	2.50649167190000
6	6.14070758730000	12.06498659680000	2.49268048480000
6	4.92407019540000	12.80675106330000	2.42459196940000
6	4.93022713770000	14.23682446990000	2.44142838130000
6	3.69848633130000	14.95430953890000	2.42677930680000
6	3.70200388240000	16.37821447910000	2.44548027250000
6	14.80388494680000	-0.00233291360000	2.46010943490000
6	14.80730607190000	1.42335211920000	2.46499326410000
6	13.57512587050000	2.13506715560000	2.46643659540000
6	13.57814762780000	3.55843030540000	2.47454505590000
6	12.34637294170000	4.26974596390000	2.49951295460000
6	12.34806546660000	5.69309261140000	2.51144863350000
6	11.11869621140000	6.40312346460000	2.57213886940000
6	11.11387027820000	7.82825377090000	2.57427243800000
6	9.88300816970000	8.53702173380000	2.65069759340000
6	9.86203026030000	9.96015942080000	2.64250745810000
6	8.60693509890000	10.65474780650000	2.64830901810000
6	8.61437166640000	12.08585150590000	2.55725856720000
6	7.37836724420000	12.79092712520000	2.53269405430000
6	7.38903904150000	14.22394144270000	2.50249207310000
6	6.16260301370000	14.94696378450000	2.47299142050000
6	6.16758733900000	16.37402035720000	2.46955224500000
6	17.26907902490000	-0.00130930960000	2.47632592260000
6	17.27357095020000	1.42508426270000	2.47477703240000
6	16.04055173400000	2.13654940240000	2.47012924240000
6	16.04257079050000	3.56125121260000	2.46512494680000
6	14.81003522590000	4.27178609370000	2.46491773570000
6	14.80911685440000	5.69727033150000	2.45974784600000
6	13.57747779580000	6.40892474330000	2.47861600550000
6	13.57182766870000	7.83328299540000	2.47995052610000
6	12.33902538400000	8.54512661130000	2.52119286160000
6	12.32835576990000	9.96970025120000	2.52234972470000
6	11.09012827850000	10.67736384800000	2.56791812300000
6	11.09001605270000	12.10357641000000	2.53459857630000
6	9.84962827730000	12.80668190060000	2.53040684720000
6	9.85628697690000	14.23533929450000	2.50606542650000
6	8.62378949650000	14.94437627760000	2.49780164910000
6	8.63179210930000	16.37561038450000	2.48261281590000
8	4.78357732020000	8.63862316850000	1.91508598940000
1	5.50032231830000	8.22089081120000	3.89659306500000

Supporting Note 7: Computational Details

In order to evaluate the strength of the cationic induced electric field in the vicinity of the catalyst from a computational point of view finite-size DFT calculations (ORCA 5.0.4) on the systems "solvated potassium + nanoflake of graphene (that represents glassy-carbon electrode)" were performed. Dipole moment and polarizability tensor obtained from these calculations were used to calculate the external electric field that would compensate the static dipole of the system (Eq. S3).

$$d_z^{total} = d_z^{static} + a_{zz} \cdot E_z \quad \text{(S3)}$$

Where d_z^{total} is the total dipole in z-direction (systems were oriented to have dipole moment in that direction), d_z^{static} is the own dipole of the system (in same direction), obtained from DFT calculation, a_{zz} is the zz-component of polarizability tensor (obtained from DFT), and E_z is the external electric field. There are numerous works that investigated K^+ cations in aqueous media (mainly different kind of MDs (MM, AIMD, MLFF) and mainly focused on statistics: average coordination numbers, average K-O distances in first shell, etc).[13-18] In order to investigate the strength of the cationic induced electric field a model of K^+ clusters with 7 surrounding water molecules ($K^+(H2O)_7$) was build.[19] We used graphene flakes of different sizes to model the infinite catalyst surface (Figure S14).

Calculations were performed with ORCA 5.0.4, D3 BJ dispersion correction and def2-TZVP basis set (smaller basis set showed significant effect on polarizability). The PBE and BHHLYP DFT functionals were tested, since it is well known that GGA functionals (including PBE) tend to delocalize the electron, while the HF (and hybrid functionals with high percentage of HF) tend to localize it. BHHLYP has thereby a 50% of HF exchange, which is one of the largest values of commonly used functionals. Thus, comparison of two extreme cases, was chosen to demonstrate the influence of electron localization (Table S2).

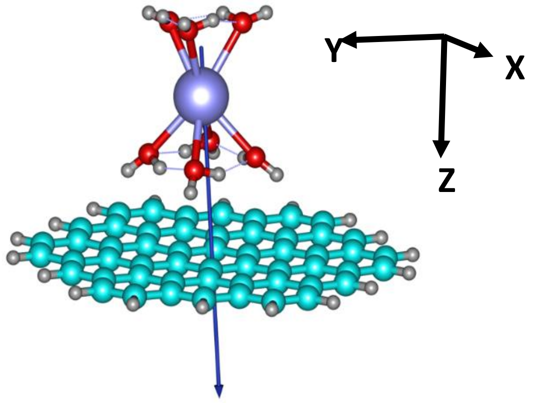

Figure S14: Model of the interaction of solvated K^+ ($K^+(H_2O)_7$) on $C_{54}H_{18}$ graphene. Colour code: Purple: K^+, cyan: C, red: O, white: H. Note, that the system is neutral, hence there is one electron in the graphene compensating the positive charge of K^+.

Table S2: Parameters for resulting induced field by ($K^+(H2O)_7$) on $C_{54}H_{18}$.

	Dipole moment / bohr^3	Polarizability tenzor / e·bohr	Electric field/ V Å^{-1}
PBE	7.94768	336.298	1.22
BHHLYP	6.98631	320.117	1.12

The electric field depends on the dipole moment and the polarizability. Therefore, different graphene flakes (with size from 42 to 110 atoms) were investigated to evaluate the effect of their size and thus the K^+-coverage/concentration on the dipole moment and the polarizability. In all cases $K^+(H2O)_7$ cluster were applied at exactly the same distance from graphene surface. Within one layer of graphene the dipole moment is almost independent from the graphene flake size (Table S3). This can be explained by the fact that already for the smallest tested flake, the valence electron of potassium is completely (based on Mullicken charges and spin densities) transferred to the nanographene, thus the charges and distances (that define dipole) stay constant. However, polarizability is strongly affected by the size of the graphene flakes (Table S3). Coordinates of calculated systems as well as pictures of them given in Table S4- 8. Applying the BHHLYP functional resulted in very similar values and are therefore not shown. We would like to underline units of all calculated parameters (Table S3), as a.u. definitions from ORCA output might be confusing.

Table S3: Dipole moment, polarizability tenzor and corresponding electric field for $K^+(H_2O)_7$ on different graphene flakes.

Graphene flake	Dipole moment / bohr^3	Polarizability tenzor / e·bohr	Electric field/ V Å^{-1}
$C_{42}H_{16}$	7.99498	282.538	1.454
$C_{54}H_{18}$	7.94768	336.298	1.215
$C_{96}H_{24}$	7.87571	477.919	0.847
$C_{110}H_{26}$	8.03929	533.521	0.774
$C_{108}H_{30}$	8.04536	532.731	0.776

Table S4: XYZ coordinates of the optimized geometries (PBE/def2-TZVP) for $K^+(H_2O)_7$–$C_{54}H_{18}$.

```
94
symmetry c1
C     6.181409000    7.854611000    5.051831000
C     6.193595000    6.417029000    5.007722000
C     7.413619000    5.741626000    4.997347000
C     7.416253000    8.568265000    5.089957000
C     6.176004000   12.128378000    5.150113000
```

C	6.180803000	10.703069000	5.137948000	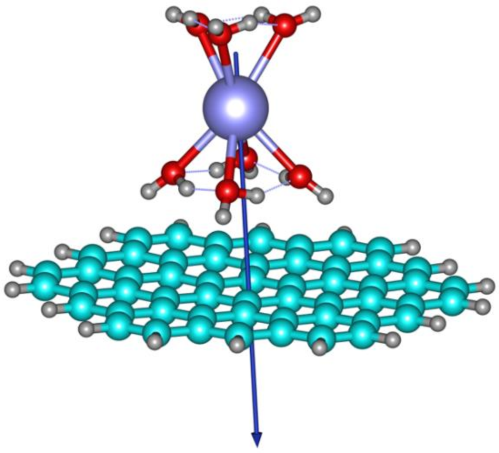
C	7.413220000	9.993529000	5.137591000	
C	7.411802000	12.844320000	5.166426000	
C	2.503472000	8.565979000	5.001549000	
C	1.249689000	12.086150000	5.008812000	
C	1.254582000	10.715999000	5.001938000	
C	2.472633000	9.971283000	5.024315000	
C	2.467535000	12.842535000	5.039378000	
C	3.702074000	7.848074000	5.010423000	
C	3.745753000	6.414884000	4.976731000	
C	4.929318000	5.733609000	4.975309000	
C	4.950450000	8.562074000	5.053005000	
C	3.707266000	12.120325000	5.075315000	
C	3.710641000	10.697741000	5.067267000	
C	4.944793000	9.986793000	5.090971000	
C	4.942343000	12.837284000	5.110595000	
C	6.176075000	16.414081000	5.079965000	
C	6.173554000	14.981168000	5.108038000	
C	7.409036000	14.269990000	5.139619000	
C	7.413230000	17.099316000	5.081392000	
C	2.486981000	14.243676000	5.031722000	
C	3.732959000	16.399923000	5.036603000	
C	3.694717000	14.974253000	5.053635000	
C	4.939220000	14.265319000	5.093096000	
C	4.923643000	17.089391000	5.049022000	
C	13.572870000	12.122228000	5.087133000	
C	13.577364000	10.752222000	5.072522000	
C	8.652250000	7.858226000	5.067091000	
C	8.649882000	6.426405000	5.021827000	
C	9.902879000	5.750604000	5.000514000	
C	9.886338000	8.573120000	5.080852000	
C	8.644644000	12.134470000	5.169498000	
C	8.649495000	10.709568000	5.152785000	
C	9.883207000	10.000493000	5.118164000	
C	9.881092000	12.851293000	5.148913000	
C	11.130312000	7.865131000	5.049697000	
C	11.093026000	6.439726000	5.013504000	
C	12.338700000	8.595230000	5.051336000	
C	11.115547000	12.140750000	5.125734000	
C	11.118488000	10.717637000	5.109147000	
C	12.359036000	9.995313000	5.077539000	
C	12.354919000	12.867473000	5.108627000	
C	8.633096000	16.423773000	5.098964000	
C	8.645137000	14.984683000	5.123482000	
C	9.876370000	14.276922000	5.130557000	
C	9.898647000	17.106491000	5.093350000	
C	11.082129000	16.424471000	5.101483000	
C	11.125922000	14.991030000	5.114151000	
C	12.324542000	14.272664000	5.108513000	
H	4.936014000	4.641319000	4.950107000	
H	2.798084000	5.871154000	4.952693000	
H	1.557371000	8.017714000	4.973172000	
H	0.311536000	10.164280000	4.977875000	
H	0.303837000	12.632561000	4.991614000	
H	1.539815000	14.788718000	5.008456000	
H	2.785331000	16.943201000	5.015261000	
H	4.923792000	18.182012000	5.036193000	

H	7.410105000	18.192621000	5.067108000
H	9.892978000	18.199069000	5.086127000
H	12.029911000	16.968576000	5.098240000
H	13.270948000	14.821068000	5.099453000
H	14.516323000	12.673762000	5.083919000
H	14.523243000	10.205857000	5.056736000
H	13.285765000	8.049826000	5.032139000
H	12.041017000	5.896898000	4.997589000
H	9.902549000	4.658297000	4.972717000
H	7.417073000	4.648648000	4.967137000
K	7.619343000	12.018409000	-0.536846000
O	7.048962000	13.839628000	-2.719897000
H	6.941659000	14.794139000	-2.855970000
H	7.905800000	13.578927000	-3.123125000
O	8.889730000	11.860869000	-3.136299000
H	9.683810000	11.513314000	-3.571976000
H	8.131331000	11.304476000	-3.424220000
O	6.242411000	11.251670000	-2.974175000
H	6.056065000	12.207812000	-3.108324000
H	5.454216000	10.760794000	-3.255031000
O	8.369740000	13.695606000	1.628637000
H	8.647603000	14.376288000	2.267304000
H	8.873371000	12.873835000	1.871239000
O	9.303428000	11.132373000	1.569101000
H	9.998762000	10.807632000	2.167488000
H	8.488811000	10.605853000	1.784028000
O	5.812526000	12.753778000	1.529741000
H	6.618476000	13.260751000	1.816557000
H	5.092351000	13.041243000	2.117577000
O	6.748046000	10.192562000	1.467851000
H	6.220512000	10.992067000	1.733032000
H	6.427980000	9.459516000	2.020990000

Table S5: XYZ coordinates of the optimized geometries (PBE/def2-TZVP) for $K^+(H_2O)_7$–$C_{42}H_{16}$.

80
symmetry c1

C	6.181409000	7.854611000	5.051831000
C	7.416253000	8.568265000	5.089957000
C	6.176004000	12.128378000	5.150113000
C	6.180803000	10.703069000	5.137948000
C	7.413220000	9.993529000	5.137591000
C	7.411802000	12.844320000	5.166426000
C	2.467535000	12.842535000	5.039378000
C	4.950450000	8.562074000	5.053005000
C	3.707266000	12.120325000	5.075315000
C	3.710641000	10.697741000	5.067267000
C	4.944793000	9.986793000	5.090971000
C	4.942343000	12.837284000	5.110595000
C	6.176075000	16.414081000	5.079965000
C	6.173554000	14.981168000	5.108038000
C	7.409036000	14.269990000	5.139619000
C	7.413230000	17.099316000	5.081392000
C	2.486981000	14.243676000	5.031722000
C	3.732959000	16.399923000	5.036603000
C	3.694717000	14.974253000	5.053635000

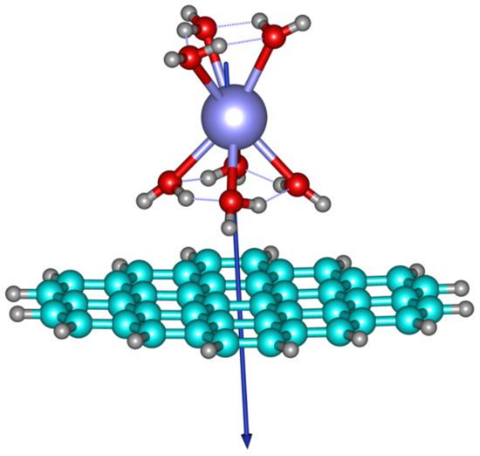

C	4.939220000	14.265319000	5.093096000
C	4.923643000	17.089391000	5.049022000
C	13.572870000	12.122228000	5.087133000
C	13.577364000	10.752222000	5.072522000
C	8.652250000	7.858226000	5.067091000
C	9.886338000	8.573120000	5.080852000
C	8.644644000	12.134470000	5.169498000
C	8.649495000	10.709568000	5.152785000
C	9.883207000	10.000493000	5.118164000
C	9.881092000	12.851293000	5.148913000
C	11.130312000	7.865131000	5.049697000
C	12.338700000	8.595230000	5.051336000
C	11.115547000	12.140750000	5.125734000
C	11.118488000	10.717637000	5.109147000
C	12.359036000	9.995313000	5.077539000
C	12.354919000	12.867473000	5.108627000
C	8.633096000	16.423773000	5.098964000
C	8.645137000	14.984683000	5.123482000
C	9.876370000	14.276922000	5.130557000
C	9.898647000	17.106491000	5.093350000
C	11.082129000	16.424471000	5.101483000
C	11.125922000	14.991030000	5.114151000
C	12.324542000	14.272664000	5.108513000
H	1.539815000	14.788718000	5.008456000
H	2.785331000	16.943201000	5.015261000
H	4.923792000	18.182012000	5.036193000
H	7.410105000	18.192621000	5.067108000
H	9.892978000	18.199069000	5.086127000
H	12.029911000	16.968576000	5.098240000
H	13.270948000	14.821068000	5.099453000
H	14.516323000	12.673762000	5.083919000
H	14.523243000	10.205857000	5.056736000
H	13.285765000	8.049826000	5.032139000
K	7.619343000	12.018409000	-0.536846000
O	7.048962000	13.839628000	-2.719897000
H	6.941659000	14.794139000	-2.855970000
H	7.905800000	13.578927000	-3.123125000
O	8.889730000	11.860869000	-3.136299000
H	9.683810000	11.513314000	-3.571976000
H	8.131331000	11.304476000	-3.424220000
O	6.242411000	11.251670000	-2.974175000
H	6.056065000	12.207812000	-3.108324000
H	5.454216000	10.760794000	-3.255031000
O	8.369740000	13.695606000	1.628637000
H	8.647603000	14.376288000	2.267304000
H	8.873371000	12.873835000	1.871239000
O	9.303428000	11.132373000	1.569101000
H	9.998762000	10.807632000	2.167488000
H	8.488811000	10.605853000	1.784028000
O	5.812526000	12.753778000	1.529741000
H	6.618476000	13.260751000	1.816557000
H	5.092351000	13.041243000	2.117577000
O	6.748046000	10.192562000	1.467851000
H	6.220512000	10.992067000	1.733032000
H	6.427980000	9.459516000	2.020990000
H	1.527779000	12.310727000	5.018417000
H	2.776527000	10.156216000	5.043160000

H	4.016896000	8.019766000	5.025085000
H	6.183042000	6.775023000	5.022044000
H	8.654127000	6.778584000	5.039338000
H	11.144174000	6.785497000	5.025234000

Table S6: XYZ coordinates of the optimized geometries (PBE/def2-TZVP) for $K^+(H_2O)_7$–$C_{96}H_{24}$.

142			
symmetry c1			
K	7.619343000	12.018409000	-0.536846000
O	7.048962000	13.839628000	-2.719897000
H	6.941659000	14.794139000	-2.855970000
H	7.905800000	13.578927000	-3.123125000
O	8.889730000	11.860869000	-3.136299000
H	9.683810000	11.513314000	-3.571976000
H	8.131331000	11.304476000	-3.424220000
O	6.242411000	11.251670000	-2.974175000
H	6.056065000	12.207812000	-3.108324000
H	5.454216000	10.760794000	-3.255031000
O	8.369740000	13.695606000	1.628637000
H	8.647603000	14.376288000	2.267304000
H	8.873371000	12.873835000	1.871239000
O	9.303428000	11.132373000	1.569101000
H	9.998762000	10.807632000	2.167488000
H	8.488811000	10.605853000	1.784028000
O	5.812526000	12.753778000	1.529741000
H	6.618476000	13.260751000	1.816557000
H	5.092351000	13.041243000	2.117577000
O	6.748046000	10.192562000	1.467851000
H	6.220512000	10.992067000	1.733032000
H	6.427980000	9.459516000	2.020990000
C	2.474645000	17.046315000	4.746049000
C	2.506814000	9.926294000	4.778991000
C	2.508638000	8.506264000	4.809456000
C	3.741546000	7.794913000	4.849095000
C	3.738306000	10.642048000	4.778855000
C	2.498470000	14.196952000	4.737532000
C	2.498356000	12.775197000	4.742300000
C	3.736318000	12.063374000	4.760518000
C	3.725573000	14.912559000	4.758381000
C	2.509747000	5.657853000	4.877929000
C	2.515362000	4.236987000	4.912083000
C	3.745271000	3.527747000	4.961889000
C	3.745050000	6.373905000	4.887964000
C	-1.202199000	10.645022000	4.712165000
C	-1.186139000	12.034229000	4.695660000
C	0.042253000	9.918771000	4.743610000
C	0.044514000	8.504182000	4.767119000
C	1.276058000	7.789627000	4.802998000
C	1.271757000	10.638093000	4.751114000
C	0.048315000	14.183104000	4.699147000
C	0.024829000	12.776984000	4.708251000
C	1.270938000	12.059017000	4.732715000
C	1.250671000	14.911790000	4.716424000
C	1.280373000	6.369111000	4.834078000

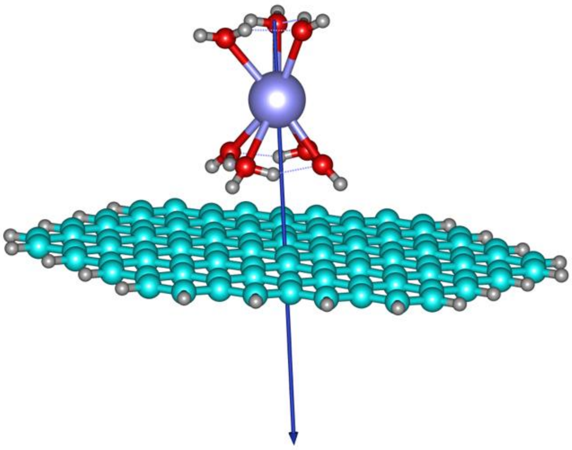

C	2.512648000	18.485482000	4.755162000
C	3.729097000	16.337114000	4.768550000
C	3.690260000	19.169107000	4.794078000
C	1.283172000	16.332389000	4.718199000
C	9.896617000	9.941708000	4.979610000
C	9.899183000	8.519302000	5.023706000
C	11.131422000	7.811399000	5.111076000
C	11.128104000	10.657347000	5.046344000
C	9.887828000	14.213285000	4.957488000
C	9.893031000	12.789668000	4.941470000
C	11.125577000	12.081645000	5.024900000
C	11.117552000	14.927796000	5.061884000
C	9.901910000	5.675922000	5.110260000
C	9.902794000	4.253142000	5.150490000
C	11.136518000	3.545461000	5.222446000
C	11.132049000	6.389723000	5.149329000
C	4.972239000	9.933999000	4.807748000
C	4.973511000	8.509925000	4.849573000
C	6.207395000	7.800123000	4.901839000
C	6.203420000	10.646093000	4.811499000
C	4.964441000	14.202729000	4.772770000
C	4.966554000	12.775819000	4.766714000
C	6.201883000	12.068303000	4.790136000
C	6.194780000	14.916873000	4.805055000
C	4.976606000	5.665212000	4.938236000
C	4.979578000	4.241387000	4.977417000
C	6.209732000	3.534477000	5.034265000
C	6.208637000	6.377682000	4.950162000
C	7.436926000	9.936920000	4.864058000
C	7.439669000	8.515330000	4.911874000
C	8.669850000	7.806057000	4.986704000
C	8.665943000	10.650878000	4.895661000
C	7.430361000	14.207050000	4.832805000
C	7.432859000	12.783101000	4.820289000
C	8.664834000	12.074655000	4.873788000
C	8.656894000	14.921411000	4.904084000
C	7.440901000	5.669882000	5.012182000
C	7.442420000	4.247232000	5.054875000
C	8.673642000	3.540389000	5.120133000
C	8.670890000	6.383101000	5.035756000
C	9.873978000	18.498452000	5.051407000
C	9.879885000	17.060222000	5.025285000
C	11.111685000	16.355108000	5.088834000
C	11.117549000	19.180493000	5.134275000
C	4.958245000	18.488288000	4.827200000
C	4.952630000	17.047013000	4.807083000
C	6.191100000	16.343441000	4.832359000
C	6.167975000	19.169042000	4.882016000
C	7.416357000	18.492424000	4.924448000
C	7.419228000	17.054450000	4.894875000
C	8.651938000	16.349416000	4.939793000
C	8.643010000	19.175483000	4.996324000
C	12.359067000	9.948005000	5.142317000
C	12.360103000	8.525071000	5.167953000
C	13.591304000	7.816695000	5.254857000
C	13.587218000	10.663496000	5.219575000
C	12.348586000	14.219147000	5.141285000

C	12.354081000	12.793444000	5.125687000
C	13.583468000	12.085115000	5.214549000
C	13.579914000	14.934468000	5.236501000
C	12.363935000	5.681832000	5.229568000
C	12.364180000	4.257837000	5.262122000
C	13.592154000	6.395608000	5.280010000
C	14.818268000	9.953327000	5.306630000
C	14.820410000	8.533090000	5.318969000
C	16.048246000	10.670810000	5.380714000
C	14.808095000	14.224388000	5.310547000
C	14.815170000	12.802725000	5.302001000
C	16.043350000	12.091905000	5.379591000
C	12.329990000	18.505373000	5.198411000
C	12.344006000	17.064089000	5.180065000
C	13.569048000	16.359220000	5.250524000
H	-2.133882000	12.577844000	4.672854000
H	-0.899070000	14.727260000	4.678832000
H	0.336203000	16.877534000	4.698230000
H	1.563866000	19.025517000	4.730510000
H	3.692196000	20.261042000	4.802217000
H	6.163690000	20.261717000	4.899218000
H	8.639247000	20.268024000	5.013701000
H	11.114924000	20.273219000	5.148814000
H	13.256345000	19.056979000	5.261626000
H	14.499648000	16.903463000	5.315197000
H	15.739994000	14.766598000	5.373572000
H	16.975946000	12.633445000	5.437888000
H	16.983750000	10.134167000	5.437690000
H	15.755423000	7.995709000	5.377050000
H	14.526841000	5.857597000	5.337502000
H	13.298853000	3.719533000	5.317027000
H	11.138384000	2.465738000	5.246865000
H	8.675207000	2.460716000	5.146676000
H	6.210522000	2.454831000	5.061909000
H	3.745363000	2.448063000	4.987992000
H	1.581474000	3.694666000	4.900131000
H	0.345793000	5.827941000	4.824337000
H	-0.890822000	7.964311000	4.757829000
H	-2.141734000	10.112520000	4.701494000

Table S7: XYZ coordinates of the optimized geometries (PBE/def2-TZVP) for $K^+(H_2O)_7$–$C_{110}H_{26}$.

158
symmetry c1

K	7.619343000	12.018409000	-0.536846000
O	7.048962000	13.839628000	-2.719897000
H	6.941659000	14.794139000	-2.855970000
H	7.905800000	13.578927000	-3.123125000
O	8.889730000	11.860869000	-3.136299000
H	9.683810000	11.513314000	-3.571976000
H	8.131331000	11.304476000	-3.424220000
O	6.242411000	11.251670000	-2.974175000
H	6.056065000	12.207812000	-3.108324000
H	5.454216000	10.760794000	-3.255031000
O	8.369740000	13.695606000	1.628637000

H	8.647603000	14.376288000	2.267304000
H	8.873371000	12.873835000	1.871239000
O	9.303428000	11.132373000	1.569101000
H	9.998762000	10.807632000	2.167488000
H	8.488811000	10.605853000	1.784028000
O	5.812526000	12.753778000	1.529741000
H	6.618476000	13.260751000	1.816557000
H	5.092351000	13.041243000	2.117577000
O	6.748046000	10.192562000	1.467851000
H	6.220512000	10.992067000	1.733032000
H	6.427980000	9.459516000	2.020990000
C	13.641967000	3.581816000	5.057009000
C	12.383453000	4.292868000	5.058896000
C	14.842187000	4.302107000	5.084511000
C	13.600621000	12.159312000	5.236777000
C	13.605138000	10.731675000	5.211136000
C	12.368182000	10.011309000	5.199884000
C	12.360088000	12.872106000	5.262615000
C	13.591179000	16.449502000	5.232671000
C	13.597237000	15.020552000	5.252264000
C	12.356253000	14.299320000	5.275999000
C	12.355126000	17.163068000	5.225490000
C	13.615924000	7.870992000	5.150327000
C	13.617302000	6.442023000	5.118994000
C	12.383425000	5.723040000	5.092615000
C	12.371730000	8.584131000	5.163604000
C	17.333961000	10.013497000	5.193220000
C	17.318926000	8.618181000	5.174341000
C	16.079281000	12.162148000	5.218801000
C	16.083303000	10.743153000	5.203679000
C	14.847365000	10.017763000	5.194994000
C	14.839685000	12.880121000	5.232893000
C	14.834494000	14.306509000	5.238684000
C	16.102561000	7.869353000	5.159716000
C	16.081624000	6.460334000	5.134886000
C	14.871437000	5.724377000	5.113400000
C	14.849497000	8.591619000	5.168824000
C	12.346094000	18.592895000	5.174707000
C	6.194945000	3.561731000	4.936659000
C	4.956386000	4.270522000	4.924267000
C	11.151855000	3.576976000	5.026683000
C	9.907965000	4.284286000	5.026767000
C	8.672783000	3.568225000	4.987760000
C	7.431430000	4.276764000	4.983590000
C	6.168301000	12.138039000	5.219713000
C	6.172503000	10.708719000	5.168209000
C	4.936715000	9.990935000	5.095150000
C	4.926529000	12.850667000	5.174879000
C	6.159045000	16.430180000	5.199335000
C	6.159682000	15.000303000	5.249010000
C	4.921926000	14.283255000	5.190309000
C	4.918240000	17.140530000	5.098806000
C	6.182096000	7.851001000	5.064411000
C	6.187949000	6.420736000	5.018265000
C	4.950561000	5.703179000	4.963517000
C	4.942498000	8.562013000	5.049345000
C	11.121444000	12.153715000	5.267028000

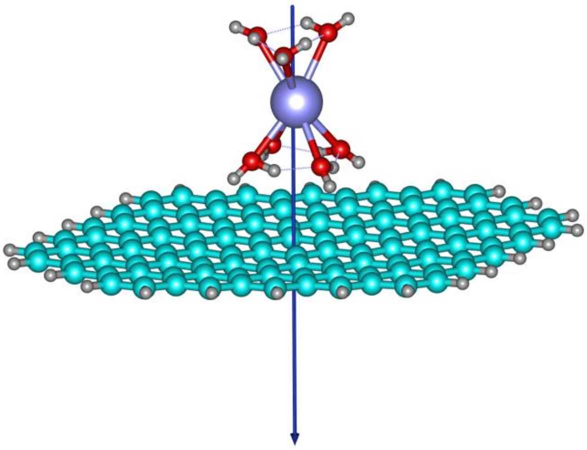

C	11.126745000	10.725116000	5.227099000
C	9.888610000	10.005360000	5.208278000
C	9.880529000	12.863929000	5.301856000
C	11.112319000	16.444935000	5.262354000
C	11.116187000	15.012146000	5.299291000
C	9.875532000	14.293288000	5.319859000
C	9.871611000	17.156329000	5.234364000
C	11.135290000	7.864452000	5.138390000
C	11.138603000	6.435184000	5.098975000
C	9.904176000	5.717746000	5.064988000
C	9.893937000	8.574844000	5.153869000
C	8.643218000	12.145437000	5.284931000
C	8.647125000	10.717318000	5.231385000
C	7.412059000	9.997473000	5.177879000
C	7.403835000	12.857857000	5.285280000
C	8.633274000	16.437020000	5.256448000
C	8.636458000	15.006084000	5.307881000
C	7.399022000	14.287289000	5.297480000
C	7.396315000	17.149227000	5.198010000
C	8.657610000	7.856958000	5.116233000
C	8.662637000	6.427684000	5.068803000
C	7.426615000	5.709870000	5.024521000
C	7.417440000	8.569121000	5.120894000
C	6.154965000	19.289933000	5.035040000
C	4.918009000	18.575066000	5.022118000
C	11.112952000	19.300063000	5.140063000
C	9.869564000	18.587379000	5.161094000
C	8.632423000	19.295500000	5.103884000
C	7.394627000	18.580482000	5.114474000
C	3.715638000	3.556389000	4.871145000
C	2.483254000	4.263975000	4.849982000
C	-1.257945000	12.111724000	4.813303000
C	-1.252821000	10.691923000	4.807354000
C	-2.506897000	12.840675000	4.748741000
C	-1.255708000	16.395030000	4.772480000
C	-1.277625000	14.986046000	4.795874000
C	-2.492250000	14.236461000	4.741424000
C	3.690877000	12.131000000	5.087256000
C	3.696850000	10.702672000	5.058156000
C	2.461565000	9.983204000	4.982029000
C	2.451599000	12.844329000	5.022723000
C	3.680929000	16.421507000	5.053582000
C	3.682471000	14.992319000	5.095435000
C	2.447529000	14.272235000	5.021036000
C	2.439210000	17.134082000	4.954048000
C	3.706134000	7.843736000	4.983046000
C	3.712376000	6.414056000	4.944470000
C	2.473428000	5.695017000	4.883491000
C	2.467885000	8.555363000	4.951432000
C	1.216793000	12.123648000	4.946379000
C	1.222291000	10.695944000	4.932521000
C	-0.014683000	9.974639000	4.861568000
C	-0.024052000	12.837615000	4.879252000
C	1.206052000	16.414386000	4.905477000
C	1.206312000	14.985132000	4.934888000
C	-0.026337000	14.263950000	4.872115000
C	-0.045798000	17.131670000	4.818177000

C	1.228886000	7.834758000	4.883201000
C	1.236854000	6.406746000	4.853969000
C	-0.007713000	8.548186000	4.841711000
C	3.675963000	19.280620000	4.930300000
C	2.442858000	18.563831000	4.901739000
C	1.187937000	19.273638000	4.812523000
C	-0.013225000	18.552566000	4.777611000
H	15.797236000	3.753229000	5.083476000
H	17.036997000	5.912390000	5.129449000
H	18.274821000	8.070739000	5.167823000
H	-0.965841000	19.101876000	4.713212000
H	-2.209085000	16.942856000	4.711006000
H	-3.446974000	14.783374000	4.688914000
H	3.716751000	2.476657000	4.847097000
H	6.197853000	2.482052000	4.910500000
H	8.676756000	2.488555000	4.961346000
H	11.157225000	2.497268000	5.002130000
H	13.656309000	2.502145000	5.034534000
H	1.175446000	20.352842000	4.772988000
H	3.672345000	20.359554000	4.882470000
H	6.154093000	20.368759000	4.984706000
H	8.631016000	20.374274000	5.052454000
H	11.113248000	20.379232000	5.097705000
H	-3.445112000	12.307417000	4.706456000
H	-2.186386000	10.150834000	4.761762000
H	-0.940591000	8.005995000	4.795152000
H	0.304218000	5.863973000	4.809389000
H	1.551069000	3.720184000	4.808480000
H	13.278998000	19.136903000	5.162646000
H	14.525331000	16.991416000	5.223264000
H	15.768739000	14.848313000	5.232784000
H	17.014201000	12.702818000	5.219689000
H	18.273387000	10.546256000	5.199993000

Table S8: XYZ coordinates of the optimized geometries (PBE/def2-TZVP) for $K^+(H_2O)_7$–$C_{108}H_{30}$.

160
symmetry c1

K	7.619343000	12.018409000	-0.536846000
O	7.048962000	13.839628000	-2.719897000
H	6.941659000	14.794139000	-2.855970000
H	7.905800000	13.578927000	-3.123125000
O	8.889730000	11.860869000	-3.136299000
H	9.683810000	11.513314000	-3.571976000
H	8.131331000	11.304476000	-3.424220000
O	6.242411000	11.251670000	-2.974175000
H	6.056065000	12.207812000	-3.108324000
H	5.454216000	10.760794000	-3.255031000
O	8.369740000	13.695606000	1.628637000
H	8.647603000	14.376288000	2.267304000
H	8.873371000	12.873835000	1.871239000
O	9.303428000	11.132373000	1.569101000
H	9.998762000	10.807632000	2.167488000
H	8.488811000	10.605853000	1.784028000
O	5.812526000	12.753778000	1.529741000

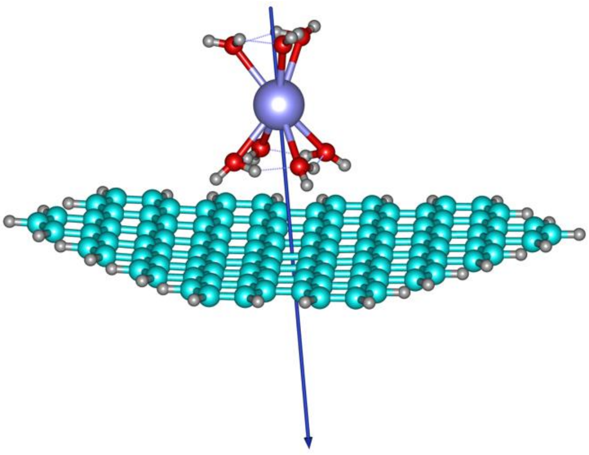

H	6.618476000	13.260751000	1.816557000
H	5.092351000	13.041243000	2.117577000
O	6.748046000	10.192562000	1.467851000
H	6.220512000	10.992067000	1.733032000
H	6.427980000	9.459516000	2.020990000
C	13.600621000	12.159312000	5.236777000
C	13.605138000	10.731675000	5.211136000
C	12.368182000	10.011309000	5.199884000
C	12.360088000	12.872106000	5.262615000
C	13.591179000	16.449502000	5.232671000
C	13.597237000	15.020552000	5.252264000
C	12.356253000	14.299320000	5.275999000
C	12.355126000	17.163068000	5.225490000
C	13.615924000	7.870992000	5.150327000
C	13.617302000	6.442023000	5.118994000
C	12.383425000	5.723040000	5.092615000
C	12.371730000	8.584131000	5.163604000
C	17.333961000	10.013497000	5.193220000
C	17.326860000	12.898873000	5.219034000
C	17.304125000	14.294298000	5.221257000
C	17.318926000	8.618181000	5.174341000
C	16.079281000	12.162148000	5.218801000
C	16.083303000	10.743153000	5.203679000
C	14.847365000	10.017763000	5.194994000
C	14.839685000	12.880121000	5.232893000
C	16.084298000	15.035045000	5.225348000
C	14.834494000	14.306509000	5.238684000
C	16.102561000	7.869353000	5.159716000
C	14.849497000	8.591619000	5.168824000
C	6.194945000	3.561731000	4.936659000
C	6.202321000	2.116124000	4.899641000
C	4.951125000	1.431150000	4.852367000
C	4.956386000	4.270522000	4.924267000
C	9.907965000	4.284286000	5.026767000
C	8.672783000	3.568225000	4.987760000
C	7.431430000	4.276764000	4.983590000
C	6.168301000	12.138039000	5.219713000
C	6.172503000	10.708719000	5.168209000
C	4.936715000	9.990935000	5.095150000
C	4.926529000	12.850667000	5.174879000
C	6.159045000	16.430180000	5.199335000
C	6.159682000	15.000303000	5.249010000
C	4.921926000	14.283255000	5.190309000
C	4.918240000	17.140530000	5.098806000
C	6.182096000	7.851001000	5.064411000
C	6.187949000	6.420736000	5.018265000
C	4.950561000	5.703179000	4.963517000
C	4.942498000	8.562013000	5.049345000
C	11.121444000	12.153715000	5.267028000
C	11.126745000	10.725116000	5.227099000
C	9.888610000	10.005360000	5.208278000
C	9.880529000	12.863929000	5.301856000
C	11.112319000	16.444935000	5.262354000
C	11.116187000	15.012146000	5.299291000
C	9.875532000	14.293288000	5.319859000
C	9.871611000	17.156329000	5.234364000
C	11.135290000	7.864452000	5.138390000

C	11.138603000	6.435184000	5.098975000
C	9.904176000	5.717746000	5.064988000
C	9.893937000	8.574844000	5.153869000
C	8.643218000	12.145437000	5.284931000
C	8.647125000	10.717318000	5.231385000
C	7.412059000	9.997473000	5.177879000
C	7.403835000	12.857857000	5.285280000
C	8.633274000	16.437020000	5.256448000
C	8.636458000	15.006084000	5.307881000
C	7.399022000	14.287289000	5.297480000
C	7.396315000	17.149227000	5.198010000
C	8.657610000	7.856958000	5.116233000
C	8.662637000	6.427684000	5.068803000
C	7.426615000	5.709870000	5.024521000
C	7.417440000	8.569121000	5.120894000
C	6.154497000	20.733530000	4.960201000
C	6.154965000	19.289933000	5.035040000
C	4.918009000	18.575066000	5.022118000
C	4.905232000	21.409474000	4.882857000
C	9.869564000	18.587379000	5.161094000
C	8.632423000	19.295500000	5.103884000
C	7.394627000	18.580482000	5.114474000
C	3.715638000	3.556389000	4.871145000
C	3.730235000	2.110117000	4.836606000
C	2.483254000	4.263975000	4.849982000
C	1.227556000	3.545431000	4.792013000
C	0.025781000	4.257729000	4.764751000
C	3.690877000	12.131000000	5.087256000
C	3.696850000	10.702672000	5.058156000
C	2.461565000	9.983204000	4.982029000
C	2.451599000	12.844329000	5.022723000
C	3.680929000	16.421507000	5.053582000
C	3.682471000	14.992319000	5.095435000
C	2.447529000	14.272235000	5.021036000
C	2.439210000	17.134082000	4.954048000
C	3.706134000	7.843736000	4.983046000
C	3.712376000	6.414056000	4.944470000
C	2.473428000	5.695017000	4.883491000
C	2.467885000	8.555363000	4.951432000
C	1.216793000	12.123648000	4.946379000
C	1.222291000	10.695944000	4.932521000
C	-0.014683000	9.974639000	4.861568000
C	-0.024052000	12.837615000	4.879252000
C	1.206052000	16.414386000	4.905477000
C	1.206312000	14.985132000	4.934888000
C	-0.026337000	14.263950000	4.872115000
C	-0.045798000	17.131670000	4.818177000
C	1.228886000	7.834758000	4.883201000
C	1.236854000	6.406746000	4.853969000
C	-0.012649000	5.682287000	4.790957000
C	-0.007713000	8.548186000	4.841711000
C	3.683741000	20.723247000	4.865951000
C	3.675963000	19.280620000	4.930300000
C	2.442858000	18.563831000	4.901739000
C	1.187937000	19.273638000	4.812523000
C	-0.013225000	18.552566000	4.777611000
H	-0.925666000	3.704384000	4.719286000

H	4.953335000	0.329916000	4.826201000
H	18.274821000	8.070739000	5.167823000
H	18.256873000	14.847296000	5.216939000
H	4.902123000	22.509799000	4.832559000
H	-0.965841000	19.101876000	4.713212000
H	-0.985271000	16.600015000	4.784500000
H	1.175446000	20.352842000	4.772988000
H	2.754136000	21.269637000	4.805193000
H	7.082097000	21.286658000	4.962947000
H	8.631016000	20.374274000	5.052454000
H	14.525331000	16.991416000	5.223264000
H	16.093967000	16.114981000	5.218640000
H	16.118219000	6.789607000	5.142273000
H	14.553975000	5.904408000	5.115254000
H	18.273387000	10.546256000	5.199993000
H	18.269138000	12.371133000	5.217496000
H	8.676756000	2.488555000	4.961346000
H	7.132711000	1.567752000	4.907581000
H	2.802532000	1.558445000	4.798851000
H	-0.955003000	6.209221000	4.764312000
H	-0.940591000	8.005995000	4.795152000
H	-0.962055000	14.800629000	4.819132000
H	1.219868000	2.465683000	4.769984000
H	-0.951349000	10.510906000	4.823169000
H	12.354612000	18.242559000	5.192347000
H	10.803654000	19.129352000	5.149192000
H	12.387874000	4.643332000	5.067885000
H	10.844455000	3.746340000	5.027476000
H	-0.957201000	12.295774000	4.834080000

References

1. Hübner, J.; Paul, B.; Wawrzyniak, A.; Strasser, P., Polymer electrolyte membrane (PEM) electrolysis of H2O2 from O2 and H2O with continuous on-line spectrophotometric product detection: Load flexibility studies. *Journal of Electroanalytical Chemistry* **2021,** *896*, 115465.
2. Lu, Z.; Chen, G.; Siahrostami, S.; Chen, Z.; Liu, K.; Xie, J.; Liao, L.; Wu, T.; Lin, D.; Liu, Y.; Jaramillo, T. F.; Nørskov, J. K.; Cui, Y., High-efficiency oxygen reduction to hydrogen peroxide catalysed by oxidized carbon materials. *Nature Catalysis* **2018,** *1* (2), 156-162.
3. Pfeifer, V.; Jones, T. E.; Velasco Velez, J. J.; Arrigo, R.; Piccinin, S.; Havecker, M.; Knop-Gericke, A.; Schlogl, R., In situ observation of reactive oxygen species forming on oxygen-evolving iridium surfaces. *Chem Sci* **2017,** *8* (3), 2143-2149.
4. Giannozzi, P.; Baroni, S.; Bonini, N.; Calandra, M.; Car, R.; Cavazzoni, C.; Ceresoli, D.; Chiarotti, G. L.; Cococcioni, M.; Dabo, I.; Dal Corso, A.; de Gironcoli, S.; Fabris, S.; Fratesi, G.; Gebauer, R.; Gerstmann, U.; Gougoussis, C.; Kokalj, A.; Lazzeri, M.; Martin-Samos, L.; Marzari, N.; Mauri, F.; Mazzarello, R.; Paolini, S.; Pasquarello, A.; Paulatto, L.; Sbraccia, C.; Scandolo, S.; Sclauzero, G.; Seitsonen, A. P.; Smogunov, A.; Umari, P.; Wentzcovitch, R. M., QUANTUM ESPRESSO: a modular and open-source software project for quantum simulations of materials. *J Phys Condens Matter* **2009,** *21* (39), 395502.
5. Perdew, J. P.; Burke, K.; Ernzerhof, M., Generalized Gradient Approximation Made Simple. *Phys. Rev. Lett.* **1996,** *77* (18), 3865-3868.

6. Prandini, G.; Marrazzo, A.; Castelli, I. E.; Mounet, N.; Marzari, N., Precision and efficiency in solid-state pseudopotential calculations. *npj Computational Materials* **2018,** *4* (72), 1-13.
7. Chen, S.; Chen, Z.; Siahrostami, S.; Kim, T. R.; Nordlund, D.; Sokaras, D.; Nowak, S.; To, J. W. F.; Higgins, D.; Sinclair, R.; Nørskov, J. K.; Jaramillo, T. F.; Bao, Z., Defective Carbon-Based Materials for the Electrochemical Synthesis of Hydrogen Peroxide. *ACS Sustainable Chemistry & Engineering* **2017,** *6* (1), 311-317.
8. Nørskov, J. K.; Rossmeisl, J.; Logadottir, A.; Lindqvist, L.; Kitchin, J. R.; Bligaard, T.; Jónsson, H., Origin of the Overpotential for Oxygen Reduction at a Fuel-Cell Cathode. *J. Phys. Chem. B* **2004,** *108* (46), 17886-17892.
9. Kulkarni, A.; Siahrostami, S.; Patel, A.; Norskov, J. K., Understanding Catalytic Activity Trends in the Oxygen Reduction Reaction. *Chem Rev* **2018,** *118* (5), 2302-2312.
10. Kelly, S. R.; Kirk, C.; Chan, K.; Nørskov, J. K., Electric Field Effects in Oxygen Reduction Kinetics: Rationalizing pH Dependence at the Pt(111), Au(111), and Au(100) Electrodes. *The Journal of Physical Chemistry C* **2020,** *124* (27), 14581-14591.
11. Li, H.; Kelly, S.; Guevarra, D.; Wang, Z.; Wang, Y.; Haber, J. A.; Anand, M.; Gunasooriya, G. T. K. K.; Abraham, C. S.; Vijay, S.; M., G. J.; Nørskov, J. K., Analysis of the limitations in the oxygen reduction activity of transition metal oxide surfaces. *Nature Catalyis* **2021,** *4*, 463–468.
12. Resasco, J.; Chen, L. D.; Clark, E.; Tsai, C.; Hahn, C.; Jaramillo, T. F.; Chan, K.; Bell, A. T., Promoter Effects of Alkali Metal Cations on the Electrochemical Reduction of Carbon Dioxide. *J Am Chem Soc* **2017,** *139* (32), 11277-11287.
13. Varma, S.; Rempe, S. B., Coordination numbers of alkali metal ions in aqueous solutions. *Biophys Chem* **2006,** *124* (3), 192-199.
14. Chen, H.; Ruckenstein, E., Hydrated Ions: From Individual Ions to Ion Pairs to Ion Clusters. *J Phys Chem B* **2015,** *119* (39), 12671-12676.
15. Ohtaki, H.; Radnai, T., Structure and Dynamics of Hydrated Ions. *Chem. Rev.* **1993,** *93*, 1157-1204.
16. Mahler, J.; Persson, I., A Study of the Hydration of the Alkali Metal Ions in Aqueous Solution. *Inorg Chem* **2012,** *51* (1), 425-438.
17. Mancinelli, R.; Botti, A.; Bruni, F.; Ricci, M. A.; Soper, A. K., Hydration of Sodium, Potassium, and Chloride Ions in Solution and the Concept of Structure Maker/Breaker. *J. Phys. Chem. B* **2007,** *111*, 13570-13577.
18. Liu, Y.; Lu, H.; Wu, Y.; Hu, T.; Li, Q., Hydration and coordination of K+ solvation in water from ab initio molecular-dynamics simulation. *J. Chem. Phys.* **2010,** *132* (12), 124503.
19. Jia, N.; Wang, P.-j.; Su, Y., Structures and spectroscopic properties of K+(H2O)n with n = 1–10 clusters based on density functional theory. *Chemical Physics Letters* **2022,** *801*, 139735.
